# Inhibition of CK1ε potentiates the therapeutic efficacy of CDK4/6 inhibitor in breast cancer

**DOI:** 10.1038/s41467-021-25700-6

**Published:** 2021-09-10

**Authors:** Fabin Dang, Li Nie, Jin Zhou, Kouhei Shimizu, Chen Chu, Zhong Wu, Anne Fassl, Shizhong Ke, Yuangao Wang, Jinfang Zhang, Tao Zhang, Zhenbo Tu, Hiroyuki Inuzuka, Piotr Sicinski, Adam J. Bass, Wenyi Wei

**Affiliations:** 1 Department of Pathology, Beth Israel Deaconess Medical Center, Harvard Medical School, Boston, MA USA; 2 State Key Laboratory for Managing Biotic and Chemical Threats to the Quality and Safety of Agro-products, School of Marine Sciences, Ningbo University, Ningbo, China; 3 Department of Liver Surgery & Liver Transplantation, State Key Laboratory of Biotherapy and Cancer Center, West China Hospital, Sichuan University, Chengdu, China; 4 Department of Medical Oncology, Dana-Farber Cancer Institute, Harvard Medical School, Boston, MA USA; 5 Department of Cancer Biology, Dana-Farber Cancer Institute and Department of Genetics, Blavatnik Institute, Harvard Medical School, Boston, MA USA; 6 Department of Pancreatic Surgery, West China Hospital, Sichuan University, Chengdu, China; 7 Division of Hematology/Oncology, Department of Medicine, Beth Israel Deaconess Medical Center, Harvard Medical School, Boston, MA USA; 8 Program in Cellular and Molecular Medicine, Boston Children’s Hospital, Boston, MA USA

**Keywords:** Kinases, Ubiquitin ligases, Ubiquitylation, Tumour-suppressor proteins

## Abstract

Although inhibitors targeting CDK4/6 kinases (CDK4/6i) have shown promising clinical prospect in treating ER+/HER2- breast cancers, acquired drug resistance is frequently observed and mechanistic knowledge is needed to harness their full clinical potential. Here, we report that inhibition of CDK4/6 promotes βTrCP1-mediated ubiquitination and proteasomal degradation of RB1, and facilitates SP1-mediated *CDK6* transcriptional activation. Intriguingly, suppression of CK1ε not only efficiently prevents RB1 from degradation, but also prevents CDK4/6i-induced *CDK6* upregulation by modulating SP1 protein stability, thereby enhancing CDK4/6i efficacy and overcoming resistance to CDK4/6i in vitro. Using xenograft and PDX models, we further demonstrate that combined inhibition of CK1ε and CDK4/6 results in marked suppression of tumor growth in vivo. Altogether, these results uncover the molecular mechanisms by which CDK4/6i treatment alters RB1 and CDK6 protein abundance, thereby driving the acquisition of CDK4/6i resistance. Importantly, we identify CK1ε as an effective target for potentiating the therapeutic efficacy of CDK4/6 inhibitors.

## Abstract (web summary)

Acquisition of CDK4/6i resistance represents a major clinical challenge. Here, the authors report that inhibition of CK1ε can prevent acquisition of CDK4/6i resistance, potentiating the therapeutic efficacy of CDK4/6i in human breast cancer.

## Introduction

Cell cycle is a series of tightly regulated molecular events that control cell division, and dysregulation of the cell cycle progression represents one of the hallmarks of tumor cells, thereby making cell cycle machinery an important target for anti-cancer therapy^1,2^. In normal conditions, three internal checkpoints, G1/S, G2-M and spindle assembly checkpoints (SAC), operate to ensure cell cycle progression in a timely and precisely regulated manner^3,4^. Cells in G1 phase receive signals and evaluate the integrity of DNA to commitment to cell cycle initiation. Mechanistically, the retinoblastoma (RB1) tumor suppressor protein binds and suppresses E2F family of transcription factors, thereby arresting cell cycle progression^5,6^. Upon receiving mitotic signals, the cyclin D-CDK4/6 kinase complex is activated, which then initiates a series of phosphorylation events to prevent RB1 from binding to E2F proteins, releasing RB1’s inhibition of cell cycle related genes to facilitate the timely cell cycle progression from G1 phase into S phase^7^. Based upon this notion, inhibitors targeting cyclin-dependent kinase 4 and 6 (CDK4/6i) have shown great success in combination with endocrine therapy in treating advanced estrogen receptor positive (ER+)/human epidermal growth factor receptor-2 negative (HER2-) breast cancer^8,9^. However, intrinsic and acquired therapeutic resistance are frequently observed in patients^10–13^, which limits their efficacy and scope of therapeutic applications.

To understand the causes and molecular mechanisms underlying drug resistance to CDK4/6i, lots of efforts had been devoted in the past few years. To this end, it has been revealed that genetic alterations of genes controlling cell cycle progression were frequently observed in breast cancer, which play crucial roles in determining the sensitivity of cancer cells to cell cycle inhibitors^10,14–16^. In addition, *PTEN* and *FAT1* loss were recently found to be involved in mediating CDK4/6i resistance in breast cancer^17,18^, and the PRMT5-MDM4 signaling axis was reported to be involved in determining the response of melanoma to CDK4/6i treatment^19^. Nonetheless, relatively little is known regarding how cancer cells adapt to drug presence upon prolonged CDK4/6i treatment. Therefore, identification of the molecular mechanisms by which cancer cells evade cell cycle checkpoint blockade would provide novel therapeutic strategy to benefit cancer treatment.

Here, we investigate the molecular events driving acquisition of CDK4/6i resistance in breast cancer cells by using CRISPR/Cas9-mediated genetic screen. In support of the critical roles they played in cell cycle progression, loss of *RB1* and upregulation of *CDK6* rank on the top hits in driving breast cancer cell evasion from CDK4/6i treatment induced cell cycle blockade. Furthermore, we show that CDK4/6i treatment can alter the protein levels of RB1 and CDK6 to confer breast cancer cells with acquisition of CDK4/6i resistance. By investigating the molecular mechanisms through which CDK4/6i treatment affects RB1 and CDK6 protein abundance, we identify CK1ε as an effective target to counteract the CDK4/6i-induced RB1 and CDK6 dysregulation. With RNA-seq and tumor xenograft models, we evaluate the therapeutic efficacy of combination of CK1i and CDK4/6i in arresting cell cycle progression and retarding tumor growth. Collectively, our results uncover a seesaw mechanism by which cancer cells evade cell cycle checkpoint blockade and establish a novel therapeutic strategy for patients with advanced breast cancers.

## Results

### CDK4/6i treatment leads to dysregulation of RB1 and CDK6 to drive acquisition of CDK4/6i resistance in breast cancer cells

**Fig. 1:**
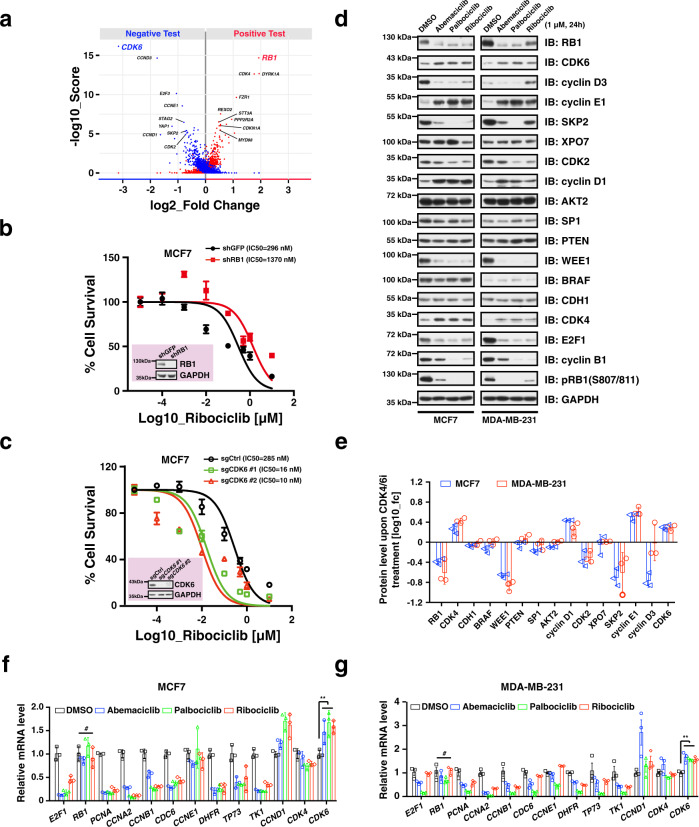
CDK4/6i treatment leads to dysregulation of *RB1* and *CDK6* to drive acquisition of CDK4/6i resistance in breast cancer. **a** Volcano plot showing drivers of CDK4/6i resistance. MCF7 and MDA-MB-231 cells were infected by a pooled CRISPR library. Cells stably expressing sgRNA were then cultured for 21 days in the presence of DMSO and ribociclib (0.5 μM), respectively. gRNAs at day 0 and day 21 were amplified by PCR and determined by Illumina deep-sequencing. The P values were based on the empirical Bayes modulated t-statistics. **b** Knocking-down of *RB1* drives resistance of MCF7 cells to ribociclib treatment (*n* = 4). **c** Knocking-out of *CDK6* sensitizes MCF7 cells to ribociclib treatment (*n* = 4). **d** Immunoblot showing the protein levels of cell cycle genes in response to CDK4/6i treatment in MCF7 and MDA-MB-231 breast cancer cell lines. **e** Quantification of protein intensity of cell cycle genes in (**d**) (*n* = 3). The quantification was performed using ImageJ (1.48 v) software. **f** RT-qPCR analysis of mRNA levels of indicated cell cycle genes in the context of CDK4/6i treatment (1 μM, 24 h) in MCF7 breast cancer cells (*n* = 3). The *P* values were calculated by Student’s t-test (two-sided). #: no significance, **P < 0.01. **g** RT-qPCR analysis of mRNA levels of indicated cell cycle genes in the context of CDK4/6i treatment (1 μM, 24 h) in MDA-MB-231 breast cancer cells (*n* = 3). The *P* values were calculated by Student’s t-test (two-sided). #: no significance, **P < 0.01. Data are presented as mean values ± SEM. The relevant raw data and uncropped blots are provided in Source Data.

To gain more insights into which genes involved in cell cycle regulation may modulate the anti-tumor efficacy of CDK4/6i in breast cancer, we performed CRISPR/Cas9-mediated genetic screen in MCF7 and MDA-MB-231 breast cancer cell lines (Supplementary Fig. 1a, Supplementary Data 1). In this screen, cells were infected with a pooled library of vectors containing both Cas9 and sgRNAs targeting components of the cell cycle. Cells were then cultured either with an inhibitor of CDK4/6 ribociclib (LEE011) or vehicle (DMSO) for 3 weeks, and then DNA was extracted and subjected to sequencing to evaluate changes in the sgRNAs in our cell population following CDK4/6i treatment. With this design, we could identify both sgRNAs enriched following CDK4/6i therapy (i.e., *RB1, CDK4, REX02 and PPP2R2A*) and sgRNAs depleted with CDK4/6i therapy (i.e., *CDK6, CCND3, E2F3 and CCNE1*). Notably, in keeping with previous reports^17,20^, we found that *CDK6* silencing was the top scoring genetic event for enhancing efficacy of CDK4/6i, and *RB1* depletion was the top hit for promoting resistance in breast cancer cells to CDK4/6i (Fig. 1a). As a proof-of-concept, focused follow-up studies silencing either *RB1* or *CDK6* led to significant alteration of drug sensitivity of MCF7 breast cancer cells to CDK4/6i (Fig. 1b, c), supporting the reliability of our in vitro screening results.

It is well known that the RB1 pathway plays a pivotal role in regulating cell cycle progression^21^. In support of this notion, exploration of data from genomics of drug sensitivity in cancer database showed that loss-of-function mutation of *RB1* had highest rank in driving CDK4/6i (palbociclib) resistance across hundreds of cancer cell lines (Supplementary Fig. 1b). In addition, cBioPortal analysis showed that genes involved in cell cycle control were frequently mutated in breast cancer, albeit in a relatively lower rate (Supplementary Fig. 1c). Notably, among those genetic alteration events whose loss enhances CDK4/6i sensitivity, *CDK6*, *CCND3*, *CCNE1* and *E2F3* were frequently amplified in the genomes of breast cancer patients (Supplementary Fig. 1d–i), whereas among the genes whose loss promotes resistance, *RB1*, *REXO2*, *PPP2R2A* and *STT3A* were mainly depleted (Supplementary Fig. 1j–o). In keeping with these results, *RB1*-deficient breast cancer cell lines exhibited strongly diminished response to CDK4/6i treatment (Supplementary Fig. 1p). Altogether, these results demonstrate that *RB1* and *CDK6* play a pivotal role in determining the cellular sensitivity of breast cancer cells to CDK4/6i.

To better understand the molecular mechanisms underlying the acquisition of drug resistance upon CDK4/6i treatment, we examined the protein levels of key cell cycle related proteins involved in regulating drug sensitivity. Of note, RB1 protein levels were dramatically reduced, while the protein abundance of cyclin E1, cyclin D1 and CDK6 were significantly accumulated following CDK4/6i treatment (Fig. 1d, e, Supplementary 1q). In keeping with the observed reduction of RB1 protein abundance, immunostaining analysis also showed decreased RB1 signals in the presence of CDK4/6i in MCF7 and MDA-MB-231 breast cancer cells (Supplementary Fig. 1r). In addition, CDK4/6i treatment led to RB1 protein reduction in a time-dependent manner in multiple breast cancer cell lines (Supplementary Fig. 2a). Moreover, ectopic expression of p16^INK4A^, an endogenous inhibitor of CDK4, and silencing of *cyclin D1* or *CDK4* also resulted in a dramatic reduction of RB1 protein abundance (Supplementary Fig. 2b-d). However, CDK4/6i treatment did not affect *RB1* mRNA levels in MCF7 and MDA-MB-231 cell lines (Fig. 1f, g), indicating that a post-translational effect was likely responsible for the rapid reduction in RB1 protein levels following therapy. In support of this notion, depletion of *CDK4* or *CDK6* shortened RB1 protein half-life (Supplementary Fig. 2e–g), accompanied by elevation of K48-linked polyubiquitination of RB1 (Supplementary Fig. 2h), indicating that suppression of cyclin D1-CDK4/6 may promote proteasomal degradation of RB1. As all of those three FDA approved CDK4/6 inhibitors (abemaciclib, palbociclib and ribociclib) displayed similar ability in driving RB1 degradation and *CDK6* upregulation (Fig. 1d–g), and ribociclib showed greatest specificity for CDK4^22^, we performed the rest of our study by using ribociclib. Taken together, these results indicate that upon CDK4/6 inhibitor treatment, RB1 protein abundance is reduced while CDK6 protein levels are accumulated, a process that may promote therapeutic resistance to these inhibitors.

### βTrCP1 mediates RB1 for proteasomal degradation upon CDK4/6i treatment

Given that suppression of cyclin D1-CDK4/6 activation arrests the cell cycle in G1 phase in a RB1-dependent manner^23^, the results we obtained above prompted us to further investigate whether RB1 is unstable in this specific cell cycle phase. In support of this notion, we found that protein levels of RB1 were significantly diminished in G1 phase throughout the cell cycle in HeLa cells (Supplementary Fig. 2i–k), which respond very well to nocodazole synchronization and is widely used in cell cycle analysis^24^. However, *RB1* transcription was not noticeably suppressed in G1 phase (Supplementary Fig. 2l), implicating that post-translational modification likely plays a major role in controlling RB1 protein oscillation throughout the cell cycle progression. In keeping with this hypothesis, RB1 protein stability was noticeably decreased in MCF7 cells in the context of serum starvation (Supplementary Fig. 2m). Collectively, these results indicate that RB1 protein may undergo proteasomal degradation in G1 phase.

**Fig. 2:**
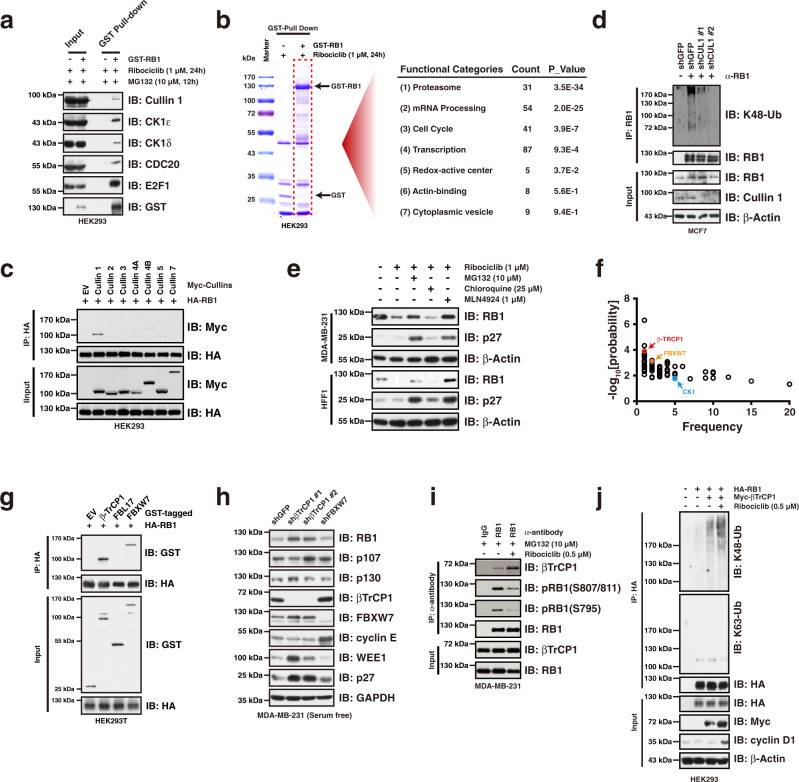
βTrCP1 mediates RB1 for proteasomal degradation upon CDK4/6i treatment. **a** Immunoprecipitation assay validating the interaction between RB1 and its interacting proteins identified by LC-MS/MS. **b** Gene functional classification of RB1-MS results showing that proteasome related proteins were significantly enriched in the GST-RB1 precipitates upon CDK4/6i treatment. Functional categories analysis was performed by the DAVID tools (https://david.ncifcrf.gov/home.jsp). The P values associated with each annotation terms inside each cluster were determined by Fisher Exact test. **c** Immunoprecipitation assay showing RB1 specifically interact with Cullin 1 in cells. **d** Knocking-down of *Cullin 1* diminished K48-linked polyubiquitination of RB1 in MCF7 cells. **e** Exposure of HFF1 and MDA-MB-231 cells with MG132 and MLN4924 blocked ribociclib treatment induced RB1 reduction. **f** Eukaryotic Linear Motif (ELM) prediction of RB1 protein showing that βTrCP1, FBXW7, and CK1 are potential RB1 interacting proteins. The prediction was performed by the tool of The Eukaryotic Linear Motif resource for Functional Sites in Proteins (http://elm.eu.org/index.html). **g** Immunoprecipitation assay showing that βTrCP1 and FBXW7 physically interacted with RB1 in cells. **h** Knocking-down of *βTrCP1*, but not *FBXW7*, led to RB1 accumulation in the context of serum starvation in MDA-MB-231 cells. **i** Immunoprecipitation assay showing that ribociclib treatment facilitated the interaction between RB1 and βTrCP1. **j** Ectopic expression of βTrCP1 elevated K48-linked polyubiquitination of RB1 in cells. Data are presented as mean values ± SEM. The relevant raw data and uncropped blots are provided in Source Data.

To identify the physiological E3(s) that is/are responsible for RB1destruction, we performed LC-MS analysis to identify the proteins interacting with RB1 in the context of CDK4/6i treatment (Supplementary Fig. 3a, Supplementary Data 2). We first validated the interaction of RB1 with some of the identified proteins in cells (Fig. 2a, Supplementary Fig. 3b), confirming the reliability of the MS results. We then performed the Gene Ontology analysis of RB1-associated proteins, which revealed a significant enrichment for proteasome-related proteins (Fig. 2b), indicating that RB1 undergoes proteasomal degradation following CDK4/6i treatment. In support of this hypothesis, CDC20 and CUL1/SKP1, critical components belonging to the APC/C and SCF complexes, two E3 ligase complexes playing crucial roles in governing cell cycle progression^4^, were identified to be associated with RB1 in the context of CDK4/6 inhibitor treatment (Supplementary Fig. 3b). We then sought to examine the potential involvement of APC/C and SCF E3 ligase complexes in the regulation of RB1 protein destruction. Although both CDC20 and Cullin 1 physically interacted with RB1 (Fig. 2a), silencing of *CDC20* did not affect RB1 protein abundance (Supplementary Fig. 3c). On the other hand, compared to other cullin family members, RB1 had the strongest interaction with Cullin 1 (Fig. 2c), which is consistent with the MS results (Supplementary Fig. 3b). Importantly, silencing of *Cullin1* diminished K48-linked polyubiquitination of RB1 (Fig. 2d), facilitating its accumulation in the context of serum starvation in MCF7 breast cancer cells (Fig. 2d, Supplementary Fig. 3d). Moreover, exposure of cells to MLN4924 which inactivates most of the Cullin-based E3 ligase complexes including SCF^25^, largely blocked ribociclib-induced RB1 degradation (Fig. 2e) and dramatically suppressed cell viability in a dose-dependent manner (Supplementary Fig. 3e). In keeping with a critical role for Cullin 1-based E3 ligase in governing RB1 protein stability, silencing *Cullin1* suppressed cell proliferation (Supplementary Fig. 3f), and enhanced the suppressive effect of CDK4/6i on cell proliferation (Supplementary Fig. 3g).

To identify the F-box protein(s) mediating RB1 degradation, we then performed a degron analysis and found βTrCP1 and FBXW7, two well-defined F-box proteins belonging to Cullin 1-based SCF E3 ligase family^26^, may serve as potential candidates (Fig. 2f). Indeed, βTrCP1 and FBXW7 were able to interact with RB1 in cells (Fig. 2g). However, knocking-down of * βTrCP1*, but not *FBXW7*, resulted in RB1 accumulation in MDA-MB-231 cells in the context of serum starvation (Fig. 2h), indicating that βTrCP1 likely serves as the physiological E3 to modulate RB1 destruction in G1 phase. In support of this notion, the RB1-βTrCP1 interaction was enhanced in the context of ribociclib treatment (Fig. 2i), but was disrupted by the RB1 hyper-phosphorylation mimic mutant, RB1-13E (Supplementary Fig. 3h). Consequently, the RB1-13E mutant showed greater stability than the RB1-13A mutant, which contains inactivating substitutions of all phospho-residues that could affect the phosphorylation-mediated conformational change of RB1 to impact RB1 interaction with its partner proteins (Supplementary Fig. 3i). Moreover, knocking-down of *βTrCP1* did not affect the protein abundance of the other two retinoblastoma-like proteins, p107 and p130 (Fig. 2h), which is consistent with the observation that βTrCP1 specifically interacted with RB1, but not p107 nor p130 in cells (Supplementary Fig. 3j). In support of the critical role of βTrCP1 in mediating RB1 for proteolytic destruction, ectopic expression of βTrCP1 elevated the K48-linked polyubiquitination of RB1 in cells (Fig. 2j). Moreover, knocking-down of *βTrCP1* suppressed HFF1 cell proliferation (Supplementary Fig. 3k), in which RB1 proteins were mainly in hypophosphorylated form and supposed to have higher affinity with βTrCP1 (Supplementary Fig. 3l), and enhanced the inhibitory effect of CDK4/6i on MCF7 breast cancer cell proliferation (Supplementary Fig. 3m). In summary, these results support the conclusion that βTrCP1 serves as the physiological F-box protein mediating RB1 for proteasomal degradation upon CDK4/6i treatment.

### CK1ε phosphorylation of RB1 promotes its degradation mediated by βTrCP1

**Fig. 3:**
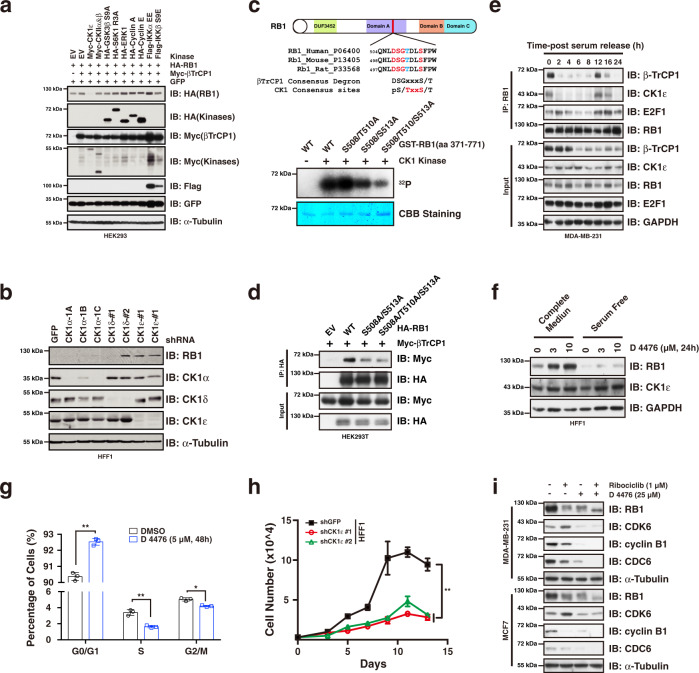
Phosphorylation of RB1 by CK1ε promotes βTrCP1-mediated degradation. **a** Ectopic expression of CK1ε led to reduced RB1 protein abundance. **b** Knocking-down of *CK1ε* resulted in RB1 accumulation in HFF1 cells. **c** In vitro kinase assay showing that CK1ε can phosphorylate RB1 at S508/T510/S513 sites. **d** Immunoprecipitation assay showing that mutation of the CK1ε phosphorylation sites of RB1 diminished its interaction with βTrCP1. **e** Immunoprecipitation assay showing that RB1 specifically interacted with βTrCP1 and CK1ε in G1 phase. MDA-MB-231 cells were serum starved for 48 h followed by serum re-addition and harvested at indicated time points. **f** Inhibition of CK1ε kinase using CK1 inhibitor D 4476 led to RB1 accumulation in a dose-dependent manner in HFF1 cells. **g** Employment of D 4476 led to increased cell population in G1 phase in HFF1 cells (*n* = 3). The *P* values were calculated by Student’s t-test (two-sided). *P < 0.05, **P < 0.01. **h** Knocking-down of *CK1ε* suppressed HFF1 cell proliferation (*n* = 4). Statistical differences were assessed by two-way ANOVA (two-sided). **: *P* < 0.01. **i** Combination of D 4476 prevented ribociclib treatment induced RB1 degradation and CDK6 accumulation in MCF7 and MDA-MB231 cells. Data are presented as mean values ± SEM. The relevant raw data and uncropped blots are provided in Source Data.

Given that a priming phosphorylation event is usually required for βTrCP1-mediated substrate recognition and destruction^27^, we then sought to investigate if this also applies for βTrCP1-mediated RB1 degradation. To this end, we transfected into HEK293T cells a panel of kinases reported previously to be involved in βTrCP1-mediated ubiquitination process. Interestingly, ectopic expression of CK1ε, but not other kinases we examined, led to reduced RB1 protein abundance (Fig. 3a, Supplementary Fig. 4a). By contrast, knocking-down of *CK1ε* resulted in RB1 accumulation in HFF1 non-transformed cells (Fig. 3b). In addition, degron analysis showed that several CK1 phosphorylation sites were present among RB1 amino acid sequence (Fig. 2f). Importantly, CK1ε was identified by the GST-RB1 MS analysis (Supplementary Fig. 3b), and further was validated by immunoprecipitation assay (Fig. 2a), implicating that CK1ε may serve as the priming enzyme. Interestingly, there is a CK1 consensus motif which is overlapped with a conserved βTrCP1 phospho-degron in RB1 amino acid sequence (Fig. 3c, up). By mutating the serine/threonine residues that construct the degron, we demonstrated that CK1ε was able to phosphorylate this phospho-degron of RB1 (Fig. 3c, bottom), and the phosphorylation was partially required for RB1 interaction with βTrCP1 in cells (Fig. 3d). Furthermore, our results showed that both CK1ε and βTrCP1 interacted with RB1 specifically in G1 phase in MDA-MB-231 cells (Fig. 3e, Supplementary Fig. 4b).

In keeping with the results that the RB1 hyper-phosphorylation mimetic 13E mutant blocked its interaction with βTrCP1 (Supplementary Fig. 3h), 13E mutant was also relatively more resistant to CK1ε-βTrCP1-mediated protein destruction (Supplementary Fig. 4c). A possible explanation would be that hyper-phosphorylation of RB1 by CDKs on all 13 Ser/Thr residues may lead to significant conformational change to disrupt RB1 interaction with βTrCP1 as it does to E2F1^28^. In keeping with the notion that CK1ε phosphorylation of RB1 promotes its destruction, treatment of HFF1 non-transformed cells with the CK1ε inhibitor, D 4476, resulted in RB1 accumulation in a dose-dependent manner (Fig. 3f), arrested the cell cycle in G1 phase (Fig. 3g), diminished cell viability and suppressed cell proliferation (Supplementary Fig. 4d–f). Additionally, knocking-down *CK1ε* mimicked the effect of the CK1ε inhibitor D 4476 on suppressing cell proliferation and enhancing the efficacy of ribociclib on cell proliferation suppression (Fig. 3h, Supplementary Fig. 4g). Importantly, addition of D 4476 blocked ribociclib-induced RB1 destruction and protected RB1 in its hypo-phosphorylated form (Fig. 3i). Altogether, these results are consistent with a model that CK1ε phosphorylates RB1 and facilitates its recognition by βTrCP1 for subsequent ubiquitination and degradation in G1 phase.

### CDK4/6 inhibition activates SP1 to confer *CDK6* upregulation

**Fig. 4:**
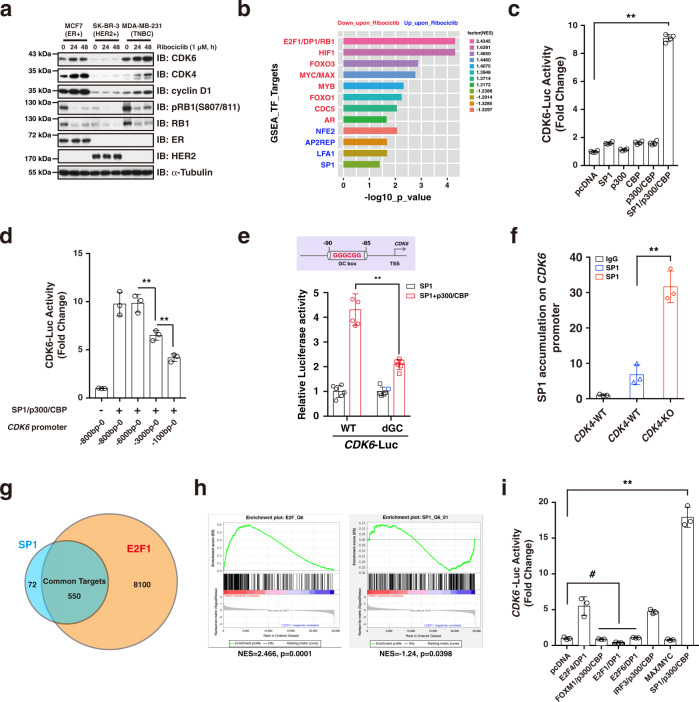
Inhibition of CDK4/6 facilitates SP1-mediated *CDK6* upregulation. **a** Ribociclib treatment led to CDK6 accumulation in a time-dependent manner in MCF7 and MDA-MB-231 cells. **b** GSEA analysis showing the enriched and suppressed transcription factors upon ribociclib treatment in MDA-MB-231 cells. The *P* values were calculated using Kolmogorov-Smirnov tests. **c** Luciferase reporter assay showing that SP1/p300/CBP complex governs *CDK6-*promoter driven luciferase expression (*n* = 4). The *P* value was calculated by Student’s t-test (two-sided). **P < 0.01. **d** Luciferase reporter assay showing that the promoter region of -600bp~0 bp of *CDK6* is responsible for SP1/p300/CBP-mediated induction of luciferase expression (*n* = 3). The *P* values were calculated by Student’s t-test (two-sided). **P < 0.01. **e** Luciferase reporter assay showing that the conserved GC box in *CDK6* promoter region is necessary for SP1/p300/CBP regulation of luciferase expression (*n* = 6). The *P* values were calculated by Student’s t-test (two-sided). **P < 0.01. **f** ChIP analysis showing that SP1 accumulation on the *CDK6* promoter region is significantly enriched in the context of *CDK4* depletion in MDA-MB-231 cells (*n* = 3). The *P* values were calculated by Student’s t-test (two-sided). **P < 0.01. **g** ChIP-seq analysis showing the overlap of target genes regulated by SP1 and E2F1. **h** GSAE analysis showing that the E2F target was reduced, while SP1_Q6_01 genes were increased in the context of ribociclib treatment in MDA-MB-231 cells. The *P* values were calculated using Kolmogorov–Smirnov tests. **i** Luciferase reporter assay showing that the SP1/p300/CBP complex plays a major role in regulating *CDK6* transcription (*n* = 3). The *P* values were calculated by Student’s t-test (two-sided). #: no significant difference, **P < 0.01. Data are presented as mean values ± SEM. The relevant raw data and uncropped blots are provided in Source Data.

As mentioned above, CDK4/6i treatment increased the expression of *CDK6* (Fig. 1f, g), which represents a major driving force underlying acquisition of CDK4/6i resistance (Fig. 1a). Therefore, finding a way to block *CDK6* upregulation is expected to prevent acquisition of drug resistance and potentiate the therapeutic efficacy of CDK4/6i. Interestingly, we observed that inhibition of CK1ε not only prevented RB1 from degradation, but also abolished ribociclib-induced CDK6 accumulation (Fig. 3i), indicating a dual effect of CK1ε inhibitor on preventing degradation of RB1 and accumulation of CDK6. We then confirmed the observation that exposure of MCF7 and MDA-MB-231 breast cancer cells to ribociclib facilitated CDK6 protein accumulation in a time-dependent manner (Fig. 4a). Given that *CDK6* transcription was elevated following CDK4/6i treatment (Fig. 1f, g), we then sought to investigate the underlying molecular mechanisms. To this end, we performed RNA-seq in ribociclib-treated MDA-MB-231 cells, and GSEA analysis showed that SP1_Q6_01 genes with promoter regions around transcription start site containing GC-box were increased upon ribociclib treatment (Fig. 4b). Furthermore, transcription factor motif analysis showed that several GC-box were presented in the promoter region (1000 bp upstream of the transcription start site) of *CDK6*. These results prompted us to hypothesize that CDK4/6 inhibition activates SP1 to confer the transcriptional activation of *CDK6*. To test this hypothesis, we cloned the promoter region of *CDK6* and generated a luciferase reporter construct. By using this system, we found that the SP1/p300/CBP complex could dramatically induce the *CDK6* promoter-driven luciferase expression (Fig. 4c), and the promoter region -600bp–0 bp was required for SP1/p300/CBP-mediated luciferase transcription (Fig. 4d). Furthermore, deleting one of the conserved GC-box within *CDK6* promoter region significantly reduced the SP1/p300/CBP-governed luciferase activity (Fig. 4e). Importantly, ChIP assay results confirmed that SP1 could bind to the GC-box within *CDK6* promoter region, which was enhanced by depleting *CDK4* (Fig. 4f).

Given that SP1 and E2F can form functional heterodimer to govern cell cycle-related gene expression^29,30^, it is interesting to ask why CDK4/6 inhibition led to E2F suppression, but SP1 activation (Fig. 4b). To try to answer this question, we then performed ChIP-seq analysis based on the existing data from GEO database. Although almost 90% of SP1 target genes were also regulated by E2F, there are nearly 10% of SP1 target genes were not subject to E2F regulation (Fig. 4g). In addition, in response to ribociclib treatment, E2F targets were dramatically suppressed, while the SP1_Q6_01 genes with promoter regions containing GC-box were enriched (Fig. 4h), indicating that the activated genes were SP1 controlled but not subject to E2F regulation. In agreement with this notion, ectopic expression of E2F/DP complex did not affect *CDK6* promoter-driven luciferase activity (Fig. 4i). Taken together, these results suggest that CDK4/6 inhibition activates SP1-mediated *CDK6* transcription.

### Suppression of CK1ε destabilizes SP1 protein and prevents ribociclib-induced *CDK6* upregulation

**Fig. 5:**
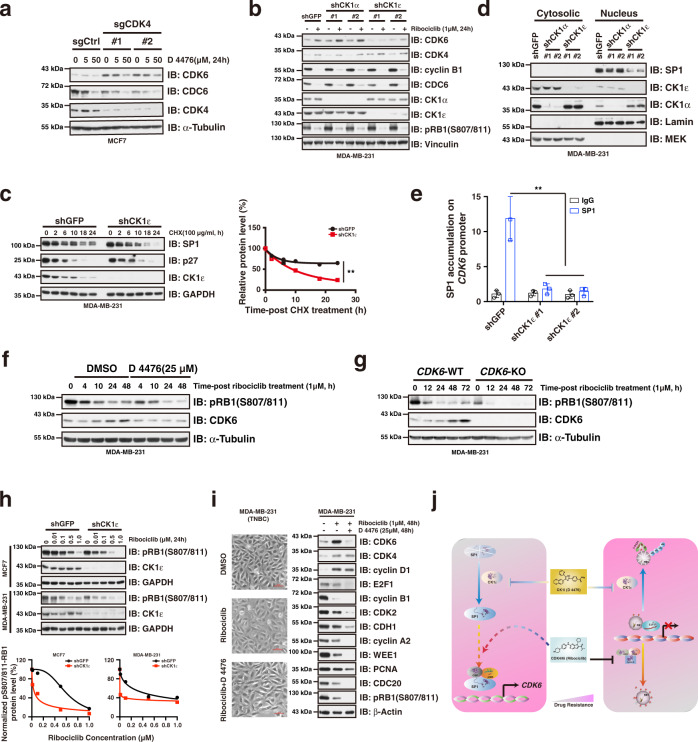
Suppression of CK1ε prevents CDK4/6i-induced *CDK6* upregulation. **a** Immunoblot showing that *CDK4*-deficiency resulted in CDK6 accumulation, which can be efficiently blocked by CK1ε kinase inhibitor D 4476. **b** Immunoblot showing that silencing of *CK1ε* abolished ribociclib-induced CDK6 accumulation. **c** Knocking-down of *CK1ε* accelerated SP1 degradation. Statistical differences were assessed by two-way ANOVA. **P < 0.01. **d** Nuclear accumulation of SP1 was significantly reduced by knocking-down of *CK1ε*. **e** Knocking-down of *CK1ε* diminished accumulation of SP1 on *CDK6* promoter region (*n* = 3). The *P* value was calculated by Student’s t-test (two-sided). **P < 0.01. **f** Treatment of MDA-MB-231 cells with D 4476 blocked CDK6 accumulation and potentiated the efficacy of ribociclib on inhibiting RB1 phosphorylation. **g** *CDK6*-deficiency potentiated the suppressing efficacy of ribociclib on RB1 phosphorylation in MDA-MB-231 cells. **h** Knocking-down of *CK1ε* sensitized MCF7 and MDA-MB-231 cells to ribociclib treatment. **i** Combination of D 4476 and ribociclib blocked CDK6 accumulation and enhanced the suppressive effect of ribociclib on cell cycle gene expression in MDA-MB-231 cells. Scale bar: 10 uM. **j** A schematic diagram showing the molecular mechanisms by which D 4476 synergizes with ribociclib to prevent acquired CDK4/6i resistance. Data are presented as mean values ± SEM. The relevant raw data and uncropped blots are provided in Source Data.

Notably, combination treatment of MCF7 and MDA-MB-231 cells with D 4476 and ribociclib not only protected RB1 from degradation, but also abolished CDK6 accumulation (Fig. 3i), indicating that the combination of D 4476 and ribociclib may prevent breast cancer cells from acquisition of CDK4/6i resistance by at least two mechanisms, namely preventing degradation of RB1 and accumulation of CDK6, respectively. This observation prompted us to investigate the molecular mechanisms by which suppression of CK1ε prevents *CDK6* induction in the context of ribociclib treatment. Consistent with the observation that D 4476 treatment blocked CDK4/6 inhibition induced CDK6 accumulation (Fig. 3i), treatment of MCF7 cells with D 4476 reduced the CDK6 protein abundance caused by *CDK4*-depletion in a dose-dependent manner (Fig. 5a). To confirm the role of D 4476 in preventing *CDK6* upregulation is on target, we then generated CK1ε depleted MDA-MB-231 cells, and results showed that silencing of *CK1ε*, but not *CK1α*, abolished ribociclib-induced CDK6 protein accumulation (Fig. 5b). In addition, knocking-down of *CK1ε*, but not *CK1α*, decreased *CDK6* mRNA levels (Supplementary Fig. 5a), while did not affect CDK6 protein stability (Supplementary Fig. 5b). Consequently, CDK6 protein abundance was decreased in the context of silencing of *CK1ε* in MDA-MB-231 and MCF7 breast cancer cells (Supplementary Fig. 5c, d). Together, these results show that CDK6 protein abundance can be manipulated by modulating CK1ε. We hypothesized that CDK4/6i treatment elevates *CDK6* transcription, while suppression of CK1ε disrupted this transactivation and thus abolished the CDK4/6i-induced *CDK6* upregulation.

To test this hypothesis, we went on to examine if there is functional crosstalk between CK1ε and SP1. Interestingly, silencing of *CK1ε* by shRNA-mediated knocking-down or employment of CK1ε inhibitor D 4476, accelerated SP1 degradation (Fig. 5c, Supplementary Fig. 5e), leading to its decreased accumulation in the nucleus in MDA-MB-231 cells (Fig. 5d), which accompanied with reduced accumulation of SP1 on the *CDK6* promoter region (Fig. 5e). In support of the critical role of CK1ε in modulating SP1 protein stability, SP1 physically interacted with CK1ε in cells (Supplementary Fig. 5f), and served as its phosphorylation substrate (Supplementary Fig. 5g). Collectively, these results suggest that ribociclib treatment enhances SP1 transcriptional activity to promote *CDK6* transcription, while inhibition of CK1ε apparently accelerates SP1 destruction to diminish its accumulation on the *CDK6* promoter region, thereby blocking the induction effect of ribociclib on *CDK6* gene transcription.

Notably, in keeping with the crucial role of *CDK6* in determining cellular sensitivity of breast cancer cells to CDK4/6i treatment, employment of CK1ε inhibitor D 4476 potentiated the suppressing efficacy of ribociclib on RB1 phosphorylation in MDA-MB-231 cells (Fig. 5f), which was similar with the results we observed in *CDK6* knockout cells (Fig. 5g), indicating a functional crosstalk between CK1ε suppression and *CDK6* down-regulation. As a proof-of-concept, we further demonstrated that either knocking-down of *CK1ε* or treatment of cells with CK1ε kinase inhibitor D 4476 significantly sensitized MCF7 and MDA-MB-231 breast cancer cells to ribociclib treatment (Fig. 5h, Supplementary Fig. 5h). Consequently, addition of D 4476 largely blocked CDK6 protein accumulation and exhibited enhanced suppressive effect on cell cycle gene expression than ribociclib single treatment (Fig. 5i, Supplementary Fig. 5i, j). Phenotypically, knocking-down of CK1ε augmented the suppressive effect of ribociclib on cell proliferation (Supplementary Fig. 5k, l). Taken together, these results demonstrate that combination of D 4476 and ribociclib is able to prevent CDK6 from accumulation caused by CDK4/6 inhibition, which provides an effective strategy to potentiate the therapeutic effect of CDK4/6i (Fig. 5j).

### D 4476 synergizes with ribociclib to arrest cell cycle progression in vitro

**Fig. 6:**
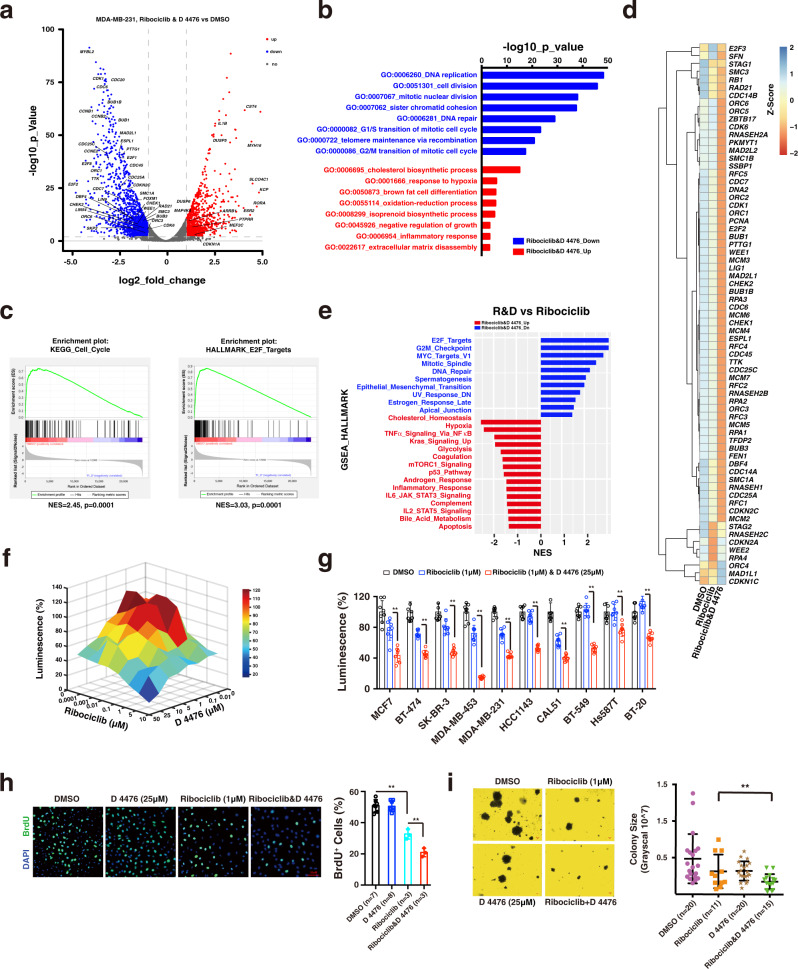
D 4476 synergizes with ribociclib to arrest cell cycle progression. MDA-MB-231 breast cancer cells were treated with DMSO, ribociclib (1 μM) and ribociclib (1 μM) plus D 4476 (25 μM) for 48 h, respectively. Total RNA was extracted, and RNA-sequencing was performed to investigate the transcription profile in response to drug treatment. **a** Volcano plot showing the up- and down-regulated genes in the ribociclib-D 4476 combination treatment group. The *P* values were calculated using t-test. **b** GO enrichment analysis showing the up-regulated and down-regulated biological processes upon ribociclib-D 4476 treatment. Statistical analysis was performed using modified Fisher’s exact tests with Benjamini–Hochberg correction. **c** GSEA analysis showing that the cell cycle pathway and E2F targets were dramatically suppressed upon ribociclib and D 4476 combination treatment. The *P* values were calculated using Kolmogorov–Smirnov tests. **d** 2D view of the cell cycle related gene expression in the context of DMSO, ribociclib, and ribociclib-D 4476 combination treatment, respectively. **e** GSEA analysis showing that, compared to the ribociclib single treatment, the upregulated and downregulated hallmarks in the ribociclib and D 4476 combination treated MDA-MB-231 cells. **f** 3D view showing that D 4476 synergized with ribociclib to suppress cell viability. Cell viability was measured by using the CellTiter-Glo Luminescent Cell Viability Assay. **g** Combination of D 4476 and ribociclib exhibited an enhanced effect on suppressing cell viability in multiple breast cancer cell lines (*n* = 8). The *P* values were calculated by Student’s t-test (two-sided). **P < 0.01. **h** BrdU assay showing that combination of D 4476 (25 μM) and ribociclib (1 μM) exhibited enhanced effect on suppressing cell proliferation. Scale bar: 10 μM. The *P* values were calculated by Student’s t-test (two-sided). **P < 0.01. **i** Combination of D 4476 with ribociclib exhibited enhanced suppressing effect on cell colony formation in MDA-MB-231 cells. The *P* values were calculated by Student’s t-test (two-sided). **P < 0.01. The relevant raw data and uncropped blots are provided in Source Data.

To understand the transcriptional response of breast cancer cells to ribociclib and D 4476 combination treatment, we then performed RNA-sequencing analysis. In keeping with the suppressive effect of ribociclib single treatment on cell cycle progression (Fig. 4h), cell cycle genes were greatly suppressed in the combination treatment cells (Fig. 6a). GO enrichment analysis showed that DNA replication and cell cycle division progression were suppressed the most dramatically (Fig. 6b). In addition, gene set enrichment analysis (GSEA) showed that the E2F target and cell cycle pathway were greatly suppressed in the combination treatment group (Fig. 6c). Notably, compared to ribociclib single treatment, the combination treatment showed similar, but greatly augmented gene response profile (Supplementary Fig. 6a–c, d, e), especially on suppressing cell cycle gene expression (Fig. 6d, e, Supplementary Fig. 6f, g), indicating that D 4476 synergizes with ribociclib in this respect. In support of this notion, combination of D 4476 and ribociclib exhibited significant synergistic effect on suppressing cell proliferation (Fig. 6f), and this synergistic effect was validated in multiple breast cancer cell lines (Fig. 6g). In keeping with these results, BrdU assay showed that cell proliferation was significantly suppressed in the combination treatment group (Fig. 6h), which was accompanied with significantly diminished cell colony size (Fig. 6i). Collectively, these results point to CK1ε an attractive target for potentiating the therapeutic efficacy of ribociclib in treating breast cancer.

### Inhibition of CK1ε overcomes CDK4/6i resistance in breast cancer cells

**Fig. 7:**
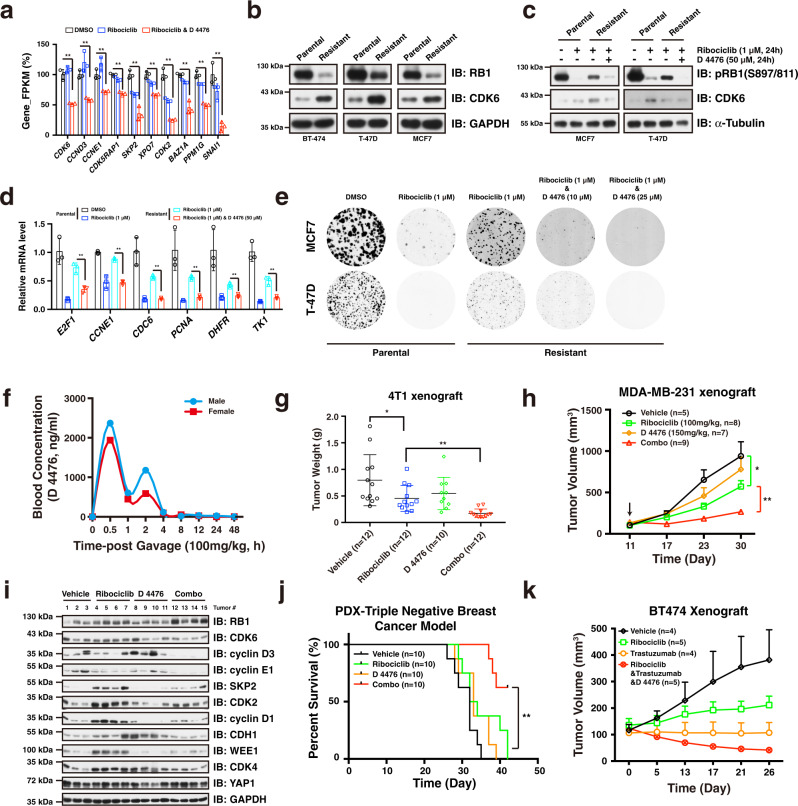
Employment of D 4476 potentiates the therapeutic efficacy of ribociclib. **a** Expression of a cohort of genes driving acquisition of CDK4/6i resistance was significantly suppressed in the context of combination treatment (*n* = 3). The *P* values were calculated by Student’s t-test (two-sided). **P < 0.01. **b** Immunoblot showing the RB1 and CDK6 protein levels in normal parental and CDK4/6i-resistant daughter breast cancer cells. **c** Immunoblot showing that addition of D 4476 potentiated the suppressing efficacy of ribociclib in CDK4/6i resistant cells. **d** RT-qPCR analysis showing that combination of D 4476 overcomes the CDK4/6i resistance in suppressing cell cycle gene transcription in CDK4/6i-resistant MCF7 cells (*n* = 3). The *P* values were calculated by Student’s t-test (two-sided). **P < 0.01. Cells were treated with ribociclib (1 μM), D 4476 (50 μM), and ribociclib-D 4476 combination, respectively, for 24 h. **e** Combination of D 4476 potentiated the efficacy of ribociclib in suppressing colony formation in MCF7 and T-47D resistant breast cancer cells. **f** Pharmacokinetic curve of CK1ε inhibitor D 4476 in vivo. A single dose of D 4476 was administrated in ICR mice and blood concentration of D 4476 at indicated time points was measured. **g** 4T1 xenograft assay showing that combination of D 4476 with ribociclib led to decreased tumor weight. The *P* values were calculated by Student’s t-test (two-sided). *P < 0.05, **P < 0.01. **h** Combination of D 4476 with ribociclib retarded MDA-MB-231 tumor growth in vivo. Statistical differences were assessed by two-way ANOVA (two-sided). *P < 0.05, **: *P* < 0.01. **i** Immunoblot analysis of MDA-MB-231 tumors treated with vehicle, ribociclib, D 4476, and Combo (ribociclib-D 4476), respectively. **j** Combination of D 4476 with ribociclib resulted in significant improvement of overall survival in triple negative breast cancer PDX model. The *P* value was calculated by log-rank test. **P < 0.01. **k** Combination of ribociclib, D 4476 and Trastuzumab retarded BT-474 tumor growth. Data are presented as mean values ± SEM. The relevant raw data and uncropped blots are provided in Source Data.

Of note, a cohort of genes contributing to CDK4/6i resistance was suppressed by the ribociclib and D 4476 combination treatment (Fig. 7a), indicating a potential role of inhibition of CK1ε in preventing acquisition of CDK4/6i resistance. We then tested this hypothesis in CDK4/6i resistant breast cancer cell lines, which was obtained by continuously being treated with CDK4/6i. In support of our model that CDK4/6i treatment promotes RB1 for proteasomal degradation and CDK6 accumulation to confer breast cancer cells with acquisition of CDK4/6i resistance, compared to parental cells, the RB1 protein levels were dramatically reduced, while CDK6 protein abundance was accumulated in resistant daughter cells (Fig. 7b). Remarkably, employment of D 4476 potentiates the efficacy of ribociclib in inhibiting RB1 phosphorylation in multiple CDK4/6i resistant breast cancer cells, accompanied with antagonizing CDK4/6i treatment induced CDK6 protein accumulation (Fig. 7c, Supplementary Fig. 7a). Consequently, addition of D 4476 re-sensitized the resistant breast cancer cells to ribociclib treatment implicated by the enhanced cell cycle gene suppression and colony formation inhibition (Fig. 7d, e). In conclusion, these results provide intriguing cellular evidence pointing to CK1ε an attractive target for preventing or overcoming CDK4/6i resistance in breast cancer cells.

### Employment of D 4476 potentiates the therapeutic efficacy of ribociclib in vivo

We then sought to evaluate the therapeutic effect of the combination strategy in vivo. To determine the tolerated dose of D 4476, we then investigated its pharmacokinetic properties in vivo. Notably, blood concentration of D 4476 reached the peak 0.5 h post gastric gavage, with a mean residence time (MRT) of 5 h in the body and oral clearance of 33% within 8 h (Fig. 7f, Supplementary Fig. 7b). Importantly, administration of D 4476 did not affect gain of weight even at a dose of 600 mg/kg (Supplementary Fig. 7c), and had minimal apparent toxic effect on the organs we examined (Supplementary Fig. 7d). Together, these results demonstrate the safety and suitable of D 4776 as a drug for using in vivo. In keeping with the complementary effect of dual targeting CK1ε and CDK4/6 in vitro, addition of CK1ε kinase inhibitor D 4476 dramatically enhanced the efficacy of ribociclib on retarding 4T1 and MDA-MB-231 tumor growth in vivo (Fig. 7g, h, Supplementary Fig. 7e, f). Notably, tumor sample analysis showed that RB1 protein abundance was slightly increased, while protein levels of CDK6, cyclin D3, cyclin E1, cyclin D1, SKP2, and CDK2 were significantly decreased in the combination treatment group (Fig. 7i). Furthermore, we utilized an RB1-positive TNBC PDX model to examine the effect of the D 4776-ribociclib combination strategy in treating human breast cancer. Of note, treatment of tumor-bearing nude mice with combination therapy resulted in a significant improvement of overall survival compared to either each single treatment (Fig. 7j, Supplementary Fig. 7g). In addition to the enhanced effect on retarding tumor growth in triple negative breast cancer models, we also observed an improved anti-cancer effect by combining ribociclib, D 4476, and trastuzumab in treating BT-474 HER2-positive breast tumor (Fig. 7k, Supplementary Fig. 7h). In summary, these results established that CK1ε inhibitor D 4476 potentiates the therapeutic efficacy of CDK4/6i ribociclib in retarding tumorigenesis, providing a novel and effective cancer treatment strategy.

## Discussion

It is widely accepted that the RB1 tumor suppressor protein serves in the central hub in the cell cycle regulation and CDK4/6i sensitivity determination^6,12^. As such, loss-of-function mutation of RB1 ranks the most important biomarker for CDK4/6i sensitivity (Fig. 1a, Supplementary Fig. 1b). RB1 mutation could be acquired, albeit at a lower rate (~5%), after exposure to CDK4/6i in the clinical setting^16,31^, establishing a feedback loop by which cancer cells evade cell cycle checkpoint inhibition. Beyond mutation, here we show that protein levels of RB1 oscillated over the course of the cell cycle, with a peak in M phase and drops when entering G1 phase (Supplementary Fig. 2k). Furthermore, we identified that the Skp/Cullin/F-box-containing (SCF) E3 ligase complex is involved in controlling the destruction of RB1 in G1 phase. Ubiquitin-mediated proteolysis plays a pivotal role in maintaining the periodic expression of cyclins, which associate with cyclin-dependent kinases (CDKs) to drive cell cycle progression^32^. These findings demonstrate that, in addition to the cyclin D-CDK4/6-mediated phosphorylation, CK1ε-βTrCP1-mediated ubiquitination also plays an important role in releasing the inhibition of RB1 on E2F transcription factors. The phosphorylation and ubiquitination modulation of RB1 serve as a double check mechanism to ensure that the E2F transactivation is fully achieved to facilitate the timely transition of cell cycle from G1 phase to S phase. Additionally, arresting cell cycle in G1 phase by utilizing CDK4/6i destabilizes RB1, which diminishes the inhibitory effect of RB1 on E2F transcription factors and may promote resistance to pharmacologic CDK4/6 blockade.

On the other hand, several lines of evidence have shown that increased CDK6 protein levels conferred breast cancer cells with resistance to CDK4/6i treatment^15,17^. Of note, genomic amplification of the *CDK6* locus was observed in breast cancer (Supplementary Fig. 1c, d). Here, we show that SP1-mediated gene expression was significantly enhanced following CDK4/6i treatment, and identify that SP1 promotes CDK6 accumulation upon ribociclib treatment. Importantly, our results establish that CK1ε functions modulate the capacity of SP1 to promote *CDK6* induction. With respect to the preferred utilization of SP1 upon ribociclib treatment, previous reports have demonstrated that phosphorylation of SP1 by cyclin B1/CDK1 represses its DNA-binding activity^33^. CDK4/6i treatment suppresses cyclin B1 expression (Fig. 1d, f, g), and thus, it is conceivable that employment of CDK4/6i would release the DNA-binding suppression of cyclin B1/CDK1 on SP1 to facilitate *CDK6* transcription. In addition, given the role of CDK4/6 in protecting cancer cells from senescence by stabilizing and activating Forkhead Box M1 (FOXM1)^34^, preventing *CDK6* expression by suppressing CK1ε is, therefore, able to accelerate cellular senescence. Indeed, a kinome-focused genetic screen identified that exposure of liver cancer cells to D 4476 could induce senescence^35^, confirming the functional crosstalk between CK1ε and CDK6.

Although the combination of CDK4/6i with endocrine therapy has led to a significant increase in the progression-free survival of patients with advanced ER+ /HER2- breast cancer^8,9^, the emergence of acquired resistance to CDK4/6i represents a major clinical challenge^10–13^. Moreover, ribociclib and palbociclib did not show significant clinical activity as a single agent. In the present study, we uncovered the molecular mechanisms by which CDK4/6i treatment leads to RB1 degradation and CDK6 accumulation, two major genetic events driving acquisition of CDK4/6i resistance. Our results also established that inhibition of CK1ε protects hypo-phosphorylated RB1 from degradation and simultaneously abolishes CDK4/6i-induced CDK6 accumulation, thereby providing a potential therapeutic strategy for breast cancer patients (Fig. 5j).

## Methods

### Cell culture and synchronization

MDA-MB-231 and MCF7 were from the A. Toker laboratory (BIDMC). CAL-51 was from the A. E. Karnoub lab (BIDMC). SK-BR-3, HCC1143, BT-474, MDA-MB-453, BT-20, and T-47D were from the P. Sicinski lab (Dana-Farbar Cancer Institute). HCC1143, BT-549, Hs587T, HEK293, HEK293T, HFF-1, MCF 10 A, WI-38, IMR-90, and HeLa were purchased from American Type Culture Collection (ATCC). Cells were maintained in a humidified incubator at 37 °C and 5% CO_2_, routinely tested, and deemed free of Mycoplasma contamination. MDA-MB-231, MCF7, HCC1143, MDA-MB-453, BT-549, Hs587T, BT-20, CAL-51, HeLa, HEK293 and HEK293T cells were cultured in DMEM supplemented with 10% fetal bovine serum. T-47D, BT-474, and SK-BR-3 cells were grown in RPMI-1640 supplemented with 10% fetal bovine serum. WI-38 and IMR-90 were maintained in Eagle’s Minimum Essential Medium supplemented with 10% fetal bovine serum. HFF-1 and MCF 10 A were cultured in DMEM/F12 media modified with 5% horse serum, 20 ng/ml EGF, 10 μg/ml Insulin, 100 ng/ml Cholera toxin, and 500 ng/ml Hydrocortisone. All medium was supplemented with 1% penicillin/streptomycin. For cell synchronization, HeLa cells with 80% confluence were treated with nocodazole (10 μM) for around 16 h, after which the suspending cells were collected and washed three times with PBS, then seeded into cell culture dishes. Cells were then harvested at indicated time points, underwent western blot, RT-qPCR, or FACS analysis.

### Plasmids

Myc-tagged Cullin 1, Cullin 2, Cullin 3, Cullin 4 A, Cullin 4B, Cullin 5, Cullin 7, βTrCP1, FBL17, FBXW7, CK1α1, CK1α2, CK1ε, CK1δ, CKIIα&β, HA-tagged CDH1, CDC20, RB1-WT, RB1-13A, RB1-13E, CBP, p300, E2F1, GSK3β S9A, S6K1 R3A, ERK1, MYC, CycA, and CycE. Flag-tagged IKKα EE, ΙΚΚβ S9E, and RB1, shRNA against Cullin 1, Cullin 2, CDK4, RB1, cyclin D1, cyclin D3, βTrCP1, CK1α, CK1δ, and CK1ε have been described previously in the papers published by the Wei laboratory^28,36^. Flag-tagged E2F4, E2F6, IRF3, DP1, MAX, FOXM1, and SP1 were purchased from OriGene. pCMV-p16 INK4A was purchased from Addgene. sgControl was a gift from Dr. W. Kaelin. sgRNAs targeting *CDK4*, *CDK6*, h*CDK6* promoter driven luciferase reporter constructs, and RB1 mutation constructs were generated in this study. Primer sequence information used in this study has been provided in Supplementary Table 1.

### Antibodies and compounds

Antibodies used in this study are as follows: anti-RB1 (#9309, 1:1000), anti-CDK2 (#2546, 1:1000), anti-CDK4 (#12790, 1:1000), anti-CDK6 (#3136, 1:1000), anti-cyclin D1 (#2978, 1:1000), anti-cyclin E1 (#4129, 1:1000), anti-SKP2 (#4358, 1:1000), anti-WEE1 (#4936, 1:1000), anti-BRAF (#9433, 1:1000), anti-Cullin 1(#4495, 1:1000), anti-pRB1(S807/811) (#9308, 1:1000), anti-AKT2 (#3063, 1:1000), anti-SP1 (#9389, 1:1000), anti-GAPDH (#2118, 1:1000), anti-cyclin A2 (#4656, 1:1000), anti-βTrCP1 (#4394, 1:1000), anti-K48-Ubiquitin (#4289, 1:1000), anti-CDC6 (#3387, 1:1000), anti-Myc-Tag (#2278, 1:2000), anti-GST (#2625, 1:1000), anti-HER2 (#2242, 1:1000), anti-Lamin A/C (#4777, 1:1000), anti-MEK1/2 (#9122, 1:1000), anti-PCNA (#2586, 1:1000), anti-p16 (#80772, 1:1000) and anti-GFP (#2555, 1:3000), anti-α-Tubulin (#3837, 1:3000), Anti-β-Actin (#3700, 1:3000) were purchased from Cell Signaling Technology. Anti-cyclin D3 (sc-6283, 1:1000), anti-XPO7 (sc-390025, 1:1000), anti-PTEN (sc-7974, 1:1000), anti-CDH1 (sc-56312, 1:1000), anti-E2F1 (sc-251, 1:1000), anti-cyclin B1 (sc-7393, 1:1000), anti-CDC20 (sc-13162, 1:1000), anti-CK1α (sc-6477, 1:1000), anti-CK1ε (sc-6471, 1:1000), anti-CK1δ (sc-6474, 1:1000), anti-p53 (sc-126, 1:1000) and anti-BrdU (sc-32323, 1:1000) were purchased from Santa Cruz. Anti-HA (H6908, 1:3000), anti-Flag (F3165, 1:3000) and anti-Vinculin (V9131, 1:10000) were purchased form Sigma. Anti-p27 (610242, 1:1000) was purchased from BD Biosciences. Anti-Thiophosphate ester antibody (ab133473, 1:1000) and anti-FBXW7 (ab109617, 1:1000) were purchased from abcam. Anti-Alexa fluor 488 polyclonal antibody (cat # A-11094, 1:1000) was purchased from Invitrogen. With respect to the drugs and kinase inhibitors used in this study, Nocodazole (M1404) was purchased from Sigma, cycloheximide (66-81-9) from Acros Organics, Palbociclib (PD-0332991, S1116) and Abemaciclib (LY2835219, S5716) from Selleckchem, MLN4924 (HY-70062), Ribociclib (LEE011, HY-15777) and D 4476 (HY-10324) from MedChemExpress, MG132 (BML-PI102-0005) from Enzo Life Science.

### Immunoblot and immunoprecipitation analysis

Cells were lysed in EBC buffer (50 mM Tris–HCl, pH 7.4; 120 mM NaCl; 0.5% NP-40) containing protease inhibitors (Complete Mini, Roche) and phosphatase inhibitors (cocktail set I and II, Calbiochem), followed by centrifuging at 12,000 g for 10 min at 4 °C. Supernatants were collected, and protein concentrations were determined by using the Bio-Rad protein assay reagent with a Beckman Coulter DU-800 spectrophotometer. Protein samples were prepared with 5x SDS loading buffer (312 mM Tris–HCl, 10% SDS, 25 mM β-mercaptoethanol, 50% glycerol, and 0.05% bromophenol blue) and boiled for 10 min. Equal amount of protein samples were then subjected to SDS-PAGE electrophoresis and transferred to methanol-activated PVDF membranes (Millipore), which were then blocked with 5% milk for 1 h at room temperature, and incubated overnight with corresponding primary antibodies at 4 °C. Membranes were washed three times with TBST buffer (20 mM Tris, 100 mM NaCl, and 0.1% Tween-20), and then incubated with secondary antibodies for 1 h at room temperature. Finally, the protein signals were developed by using ECL western blotting substrate (Thermo Pierce) and visualized by western film processor. For immunoprecipitation analysis, cell lysates with 1000 μg of total protein were incubated with primary antibody-conjugated protein A/G beads, or HA-/Flag-conjugated agarose beads overnight or 6 h at 4 °C, respectively. The recovered immunoprecipitates were washed four times with NETN buffer (20 mM Tris, pH 8.0; 100 mM NaCl; 0.5 mM EDTA and 0.5% NP-40), resolved by SDS–PAGE and immunoblotted with indicated antibodies.

### Liquid chromatography and tandem mass spectrometry

GST-tagged RB1 was ectopically expressed in HEK293 cells. Cells were lysed 36 h post transfection, and protein concentrations were measured as described above. In total 1000 μg of total protein were incubated with Glutathione sepharose 4B (GE Healthcare Dharmacon, catalog number: 17-0756-01) for 6 h at 4 °C, followed by washing four times with NETN buffer (100 mM NaCl; 20 mM Tris–HCl, pH 8.0; 0.5 mM EDTA; 0.5% NP-40). The proteins were then eluted from the beads with glutathione and precipitated by using Trichloroacetic acid (TCA) before being subjected to do mass spectrometry analysis. Cluster analysis was performed by using DAVID tool^37^. Source data has been provided in Supplementary Data 2.

### RNA isolation and quantitative reverse-transcription PCR

Total RNA was extracted from cultured breast cancer cells using Trizol (Invitrogen) and reverse-transcribed into cDNA by using iScript^TM^ reverse transcription supermix (BIO-RAD) following manufacturer’s instructions. Quantitative PCR reactions were performed with PowerUp^TM^ SYBR^TM^ Green Master Mix (ThermoFisher Scientific), and amplification of the desired products were monitored and recorded using CFX96^TM^ Real-Time PCR Detection System (BIO-RAD). The sequences of the primers used for quantitative reverse-transcription PCR are provided in Supplementary Table 2.

### Luciferase assay

Luciferase assay was performed as previously described^38^. In brief, HEK293T cells were cultured in 24-well plate, harvested and lysed 36 h post-transfection by adding 150 μl of Gly-gly buffer (25 mM Gly-gly, pH 7.8; 15 mM MgSO_4_; 4 mM EGTA, pH 7.8; 1 mM DTT and 1% Triton X-100) per well. Aliquot of 25 μl lysate per well was transferred to a black 96-well plate accordingly, followed by adding 25 μl of the luciferase assay mixture (20 mM Gly-gly, pH 7.8; 12 mM MgSO_4_; 3 mM EGTA, pH 7.8; 0.2 mM potassium phosphate, pH 7.8; 2 mM ATP; 1.5 mM DTT and 1.25 mg/ml firefly luciferin). Fluorescence intensity was measured by using Epoch Microplate Spectrophotometer (BioTek).

### CRISPR/Cas9-mediated screen

For the design of the CRISPR library, 5935 gRNAs targeting 1187 human cell cycle-related genes were designed and cloned into LentiCRISPRv2.1. The genome CRISPR library was introduced into MCF7 and MDA-MB-231 cells by lentiviral infection, followed by selecting with puromycin. Cells stably expressing gRNA were then cultured for 21 days in the presence of DMSO and ribociclib (0.5 μM) treatment, respectively. gRNA barcodes from samples at day 0 and day 21 were amplified by PCR and determined by Illumina deep-sequencing. The cell cycle-related gene list was decided by combination of GO_CELL CYCLE and GSEA_KEGG/HALLMARKS_CELL_CYCLE. Source data has been provided in Supplementary Data 1.

### Protein purification and in vitro kinase assay

pGEX4T1-GST and pGEX4T1-GST-RB1 were transformed into BL21 competent cells, respectively. The transformed cells were incubated in 4 ml of LB medium containing 200 μg/ml ampicillin and incubated at 37 °C with a shaker for 8 h, followed by transferring the incubated 4 ml of LB medium into 400 ml LB medium containing ampicillin. IPTG (final concentration of 400 μM) was added when the OD600 ranks 0.4–0.6 and continued incubating at 16 °C with a shaker for overnight. The bacteria pellets were collected, followed by adding 10 ml of lysis buffer (20 mM Tris–HCl pH 8.0, 2 mM EDTA, 2 mM DTT, 300 mM NaCl, protease, and phosphatase inhibitors), sonicating to disrupt cells, and centrifuging to collect the supernatant, incubating with Glutathione sepharose 4B beads for 3 h at 4 °C. Beads were washed with NETN buffer for 5 times and resuspended in 50 mM Tris–HCl pH 8.0. For Flag-SP1 purification, pcDNA-cFlag-SP1 plasmids were transfected into HEK293T cells, followed by being immunoprecipitated with anti-Flag M2 magnetic beads 36 h post transfection. Beads were washed with high salt wash buffer (0.1% SDS, 0.1% Triton X-100, 300 mM NaCl, 2 mM EDTA and 20 mM Tris–HCl pH 8.0) and resuspend in 50 mM Tris–HCl pH 8.0. In vitro kinase assay was performed as previously described^39^. Briefly, the purified proteins were incubated with ATPγS and CK1ε kinase in the kinase reaction buffer (50 mM Tris–HCl; 10 mM MgCl_2_; 0.1 mM EDTA; 2 mM DTT and 0.01% Brij 35, pH 7.5) for 30 min at room temperature, followed by adding PNBM and continue incubating for 2 h at RT. The reaction was then terminated by adding 5x SDS loading buffer and boiled for 10 min. Samples were then subjected to western blot analysis and immunoblotted with anti-Thiophosphate ester antibody (ab133473).

### Cell viability assay

For cell viability assay, cells were seeded into 96-well plates (1000 cells per well) on day 1, followed by treating with indicated compounds for 72 h. The cell viability was then measured by using the CellTiter-Glo (Promega) according to manufacturer’s instructions. Briefly, the culture medium was changed with 50 μl of fresh one, and the plates and its contents were equilibrated at room temperature for 30 min. In total 50 μl of CellTiter-Glo Reagent was then added into each well, and contents were mixed for 2 min on an orbital shaker to induce cell lysis. The plates were allowed to incubate at room temperature for 10 min, and luminescence was recorded with Epoch Microplate Spectrophotometer (BioTek).

### Fluorescence-activated cell sorting (FACS) analysis

Flow cytometry was applied to assess the cell cycle progression in this study. In brief, cells with indicated treatment were harvested and washed once with PBS. Cell number was counted and approx 10^6^ cells were fixed with 70% ethanol overnight at 4 °C, centrifuged, and ethanol was decanted thoroughly, followed by washing once with cold PBS. The cell pellets were resuspended in 1 ml of PI staining solution (0.1% Triton X-100; 10 μg ml^−1^ PI; 100 μg ml^−1^ DNase-free RNase; 1 mM EDAT and 2% FBS in PBS) and incubated in the dark at room temperature for 30 min. Samples were transferred to Beckman Coulter CytoFLEX LX and fluorescence was recorded by CytExpert (v2.3). Data was analyzed by FlowJo software (v10.4.0). Data were derived from all cells without gating strategy applied.

### Chromatin immunoprecipitation

ChIP assay was performed using EpiQuik Chromatin Immunoprecipitation Kit (Epigentek, P-2002) according to manufacturer’s instructions. Briefly, cells were harvested and cross-linked with 1% formaldehyde at room temperature for 20 min. DNA was sheared into fragments of 100–1000 bp with a sonicator. Supernatants were then transferred into a 96-well plate pre-coated with SP1 antibody, followed by washing and recovering the precipitated DNA complex. Target promoters were analyzed using quantitative reverse-transcription PCR (RT-qPCR). Primer sequence used for RT-qPCR has been provided in Supplementary Table 2.

### BrdU labeling and immunostaining

MDA-MB-231 cells were seeded on coverslips in six well plates (5 × 10^4^ cells per well) and grow for 12 h before being treated with DMSO, ribociclib (1 μM), D 4476 (25 μM), and ribociclib-D 4476 combination, respectively. The existing cell culture medium was removed and replaced with 10 μM BrdU labeling solution (10 μM BrdU in cell culture medium) 24 h post-treatment with indicated compounds, followed by incubating at 37 °C for 6 h. The BrdU labeling solution was removed from the cells and washed five times with PBS. Cells were then fixed with 4% formaldehyde for 15 min and permeabilized with 0.2% Triton X-100 for 20 min at room temperature successively. Cells were incubated with 2 N HCl for 30 min at room temperature to achieve denature of the DNA, followed by removing the HCl and neutralizing with 0.1 M phosphate/citric acid buffer pH 7.4 for 30 min at room temperature. Cells were then washed twice with 0.2% Triton X-100, blocked with 5% milk, and incubated with primary antibody against BrdU overnight at 4 °C. The solution was decanted and cells were washed three times with TBST before being incubated with secondary antibody conjugated with Alexa fluor 488 at room temperature for 1 h. Finally, cells were washed three times with TBST, mounted with coverslip with a drop of Thermo ProLongTM Gold Antifade Mountant with DAPI.

### RNA sequencing and gene annotation

MDA-MB-231 cells were treated with DMSO, ribociclib (1 μM) and ribociclib (1 μM)-D 4476 (25 μM) combination for 48 h. Total RNA was extracted using TRIzol reagent (ThermoFisher) and quantified by a NanoDrop ND-2000 instrument. In total 5 μg of the RNA was used to construct the sequencing cDNA library in the following steps: two rounds of purification were conducted using oligo(dT) magnetic beads (rRNA removed) to enrich Poly(A) RNA. The poly(A) RNA was fragmented into small pieces using divalent cations under high temperature and reverse-transcribed to create the first cDNA strand. U-labeled second-stranded cDNA was then synthesized with E. coli DNA polymerase I, RNase H, dUTP and the first strand as template. After that, an A-base is added to the blunt ends of both strands and ligated to the indexed adapters and size selection was performed with AMPureXP beads. The ligated products are amplified with PCR according to the following protocol: initial denaturation at 95 °C for 3 min; 8 cycles of denaturation at 98 °C for 15 s, annealing at 60 °C for 15 s, and extension at 72 °C for 30 s; then final extension at 72 °C for 5 min. The average insert size for the final cDNA library was 300 bp (±50 bp). The 150 bp paired-end sequencing was performed on an Illumina Hiseq 4000 (LC-BIOTECHNOLOGIS (HANGZHOU) CO., LTD). Raw sequencing reads were quality trimmed and aligned to reference human genome hg38 using HISAT2 (2.0.4) package. The transcript abundances for each sample was estimated with StringTie (1.3.4). All transcriptomes from each sample were merged to reconstruct a comprehensive transcriptome and subjected to StringTie (v1.3.4) and Ballgown (2.24.0) to estimate the expression levels of all transcripts. The FPKM value for mRNA expression levels were calculated with Ballgown. The differentially expressed mRNAs and genes were filtered with log2 (fold change) ≥ 1 or log2 (fold change) ≤ −1 and with statistical significance (*P* value < 0.05) by R package-edgeR (v3.34.0). GO analysis for enriched ‘biological process’ terms were performed using the web tool DAVID bioinformatics Database v6.8 (https://david.ncifcrf.gov/tools.jsp). For GSEA analysis, we used the GSEA tool v4.1.0, with the MSigDB v7.4 ‘Hallmarks’ and ‘TFT_Legacy’ gene sets respectively for ‘hallmark signatures’ and ‘transcription factor targets’; the ‘classic’ method was used for calculating enrichment scores. Scatter plots, volcano plots, and heatmap were plotted with the differentially expressed genes in R.

### Pharmacokinetics-pharmacodynamic analysis

Pharmacokinetic of D 4476 was determined to evaluate its toxicity in vivo. Briefly, 18 ICR mice (six-week-old) were divided into four groups and each group has the same number of male and female mice. Mice in each group were given with different concentration of D 4476 by gastric gavage (D 4476 was dissolved in methyl cellulose, 15 cP), two mice with 30 mg/kg, two with 60 mg/kg, four with 400 mg/kg, and 10 with 600 mg/kg. The health and vitality were observed for seven days and the weights were measured at day 0. Day 1, day 3, day 5, and day 7. The organ/weight ratios were calculated after the mice were sacrificed. A total of 18 ICR mice (six-week-old) were oral administrated with 100 mg/kg of D 4476, and blood samples of every two mice (one male and one female) were collected at 0, 0.5, 1, 2, 4, 8, 12, 24, and 48 h post gastric gavage. The plasma was extracted and the drug concentration was determined using the LC–MS/MS.

### Xenograft assay

Animal studies were approved by Dana-Farber Cancer Institute Institutional Animal Care and Use Committee (IACUC; protocol number 11-009), Beth Israel Deaconess Medical Center Institutional Animal Care and Use Committee (IACUC; protocol number 043-2015), and performed in accordance with guidelines established by NIH Guide for the care and use of laboratory animals. All animals were housed on a 12 light/12 dark cycle, temperature 70° ± 5 F, and humidity 40–60%. 4T1, MDA-MB-231 and BT-474 cell lines were detected as pathogen free and cultured in DMEM and RPMI-1640 with 10% FBS, respectively. For 4T1 and MDA-MB-231 xenograft studies, 4T1 (1 × 10^5^) and MDA-MB-231 cells (2 × 10^6^) were suspended in 150 μl of DMEM medium and subcutaneously injected into the 6-week-old nude female mice. Mice were randomly divided into 4 groups, and treated daily with vehicle (0.5% methyl cellulose), ribociclib (100 mg/kg), D 4476 (150 mg/kg), and ribociclib-D 4476 combination by gastric gavage, respectively, when the tumor size reached 100 mm^3^. Tumor size was observed and measured as indicated. For TNBC PDX model, patient-derived triple negative breast cancer tissues were implanted into 6-week-old nude female mice, followed by treating with indicated therapeutic strategy when tumors reach 200 mm^3^. Mice were sacrificed when tumor volume reaches 3000 mm^3^ and deemed as death. Survival curve was plotted by using Graphpad Prism 7. For BT-474 xenograft model, the cells were harvested and resuspended in serum-free medium, mixed with an equal amount of Matrigel (BD Biosciences). Mice were injected with 2 million cells per injection with two distinct injections in the flank of each mouse. Then the mice were randomly grouped, and treatment was started when the tumors’ size reached 100–150 mm^3^. Each cohort included at least 4 tumors. Tumor sizes were monitored twice weekly and volumes were calculated with the formula: (mm^3^) = length_Xwidth_XwidthX_0.5. D 4476 and ribociclib were dissolved in 0.5% hydroxypropyl methylcellulose with 0.4% Tween 80. D 4476 was dosed as 150 mg/kg daily via garage, ribociclib was given as 100 mg/kg daily via garage, and Trastuzumab (Herceptin) was dosed as 10 mg/kg twice a week via intra-peritoneum (IP).

### Statistics and reproducibility

Statistical analyses were performed as described in the figure legend for each experiment. Results were represented as mean ± s.e.m unless otherwise noted in the figure legend. Statistical tests were selected based on appropriate assumptions with respect to data distribution and variance characteristics. Differences were considered statistically significant at *P* ≤ 0.05. No statistical methods were used to predetermine sample size. All data shown are representative of two or more independent experiments with similar results, unless indicated otherwise.

### Reporting summary

Further information on research design is available in the Nature Research Reporting Summary linked to this article.

## Supplementary information

Supplementary Information

Description of Additional Supplementary Files

Supplementary Data 1

Supplementary Data 2

Reporting Summary

## Data Availability

The RNA sequencing data generated in this study have been deposited into the Gene Expression Omnibus Database under accession code GSE177054. The CRISPR screen data generated in this study is provided in Supplementary Data 1. The MS data generated in this study is provided in Supplementary Data 2. The human cancer data (Supplementary Fig. 1c–o) were derived from cBioPortal for cancer genomics (https://www.cbioportal.org). The data for Supplementary Figure 1b, p were achieved from Genomics of Drug Sensitivity in Cancer database (https://www.cancerrxgene.org). Publicly available E2F1 and SP1 ChIP-seq datasets from MCF7 cells (Fig. 4g) were obtained from the GEO database (GSE92014: https://www.ncbi.nlm.nih.gov/geo/query/acc.cgi and GSE31477: https://www.ncbi.nlm.nih.gov/geo/query/acc.cgi). These datasets were aligned to the hg38 genome using the Bowtie 2v2.2.9. Peak calling was performed using MACS2 v2.1.1 with default parameters except ‘-q 0.001’. The GREAT software v4.0.4 (http://great.stanford.edu/public/html/) was used to assign peaks to genes (within 500 bp up- or downstream of the TSS). DAVID bioinformatics Database v6.8 can be accessed through https://david.ncifcrf.gov/tools.jsp. Source data are provided with this paper.

## Source data

Source Data
