# Supplementary material for: Inhibition of CK1ε potentiates the therapeutic efficacy of CDK4/6 inhibitor in breast cancer
Source: Nat Commun. 2021 Sep 10;12:5386. doi: 10.1038/s41467-021-25700-6 (PMC8433397; doi:10.1038/s41467-021-25700-6)
Supplement: Supplementary file 1 — Supplementary Information [file 41467_2021_25700_MOESM1_ESM.pdf]

Supplementary Information

Inhibition of CK1ε potentiates the therapeutic efficacy of CDK4/6 inhibitor in breast cancer

Fabin Dang[1, 9], Li Nie[1, 2, 9], Jin Zhou[3, 4, 9], Kouhei Shimizu[1], Chen Chu[5], Zhong Wu[4, 6], Anne Fassl[5], Shizhong Ke[7], Yuangao Wang[8], Jinfang Zhang[1], Tao Zhang[1], Zhenbo Tu[1], Hiroyuki Inuzuka[1], Piotr Sicinski[5], Adam J. Bass[4#], Wenyi Wei[1#]

1. Department of Pathology, Beth Israel Deaconess Medical Center, Harvard Medical School, Boston, MA, USA
2. State Key Laboratory for Managing Biotic and Chemical Threats to the Quality and Safety of Agro-products, School of Marine Sciences, Ningbo University, Ningbo, China
3. Department of Liver Surgery & Liver Transplantation, State Key Laboratory of Biotherapy and Cancer Center, West China Hospital, Sichuan University, Chengdu, China.
4. Department of Medical Oncology, Dana-Farber Cancer Institute, Harvard Medical School, Boston, MA, USA.
5. Department of Cancer Biology, Dana-Farber Cancer Institute and Department of Genetics, Blavatnik Institute, Harvard Medical School, Boston, MA, USA.
6. Department of Pancreatic Surgery, West China Hospital, Sichuan University, Chengdu, China.
7. Division of Hematology/Oncology, Department of Medicine, Beth Israel Deaconess Medical Center, Harvard Medical School, Boston, MA, USA
8. Program in Cellular and Molecular Medicine, Boston Children's Hospital, Boston, MA, USA.
9. These authors contributed equally to this work.

# Correspondence
A. B.: adam_bass@dfci.harvard.edu; 450 Brookline Ave, Boston, MA 02215
W.W.: wwei2@bidmc.harvard.edu; E/CLS-637, 3 Blackfan Circle, Boston, MA 02215

Sup. Fig 1

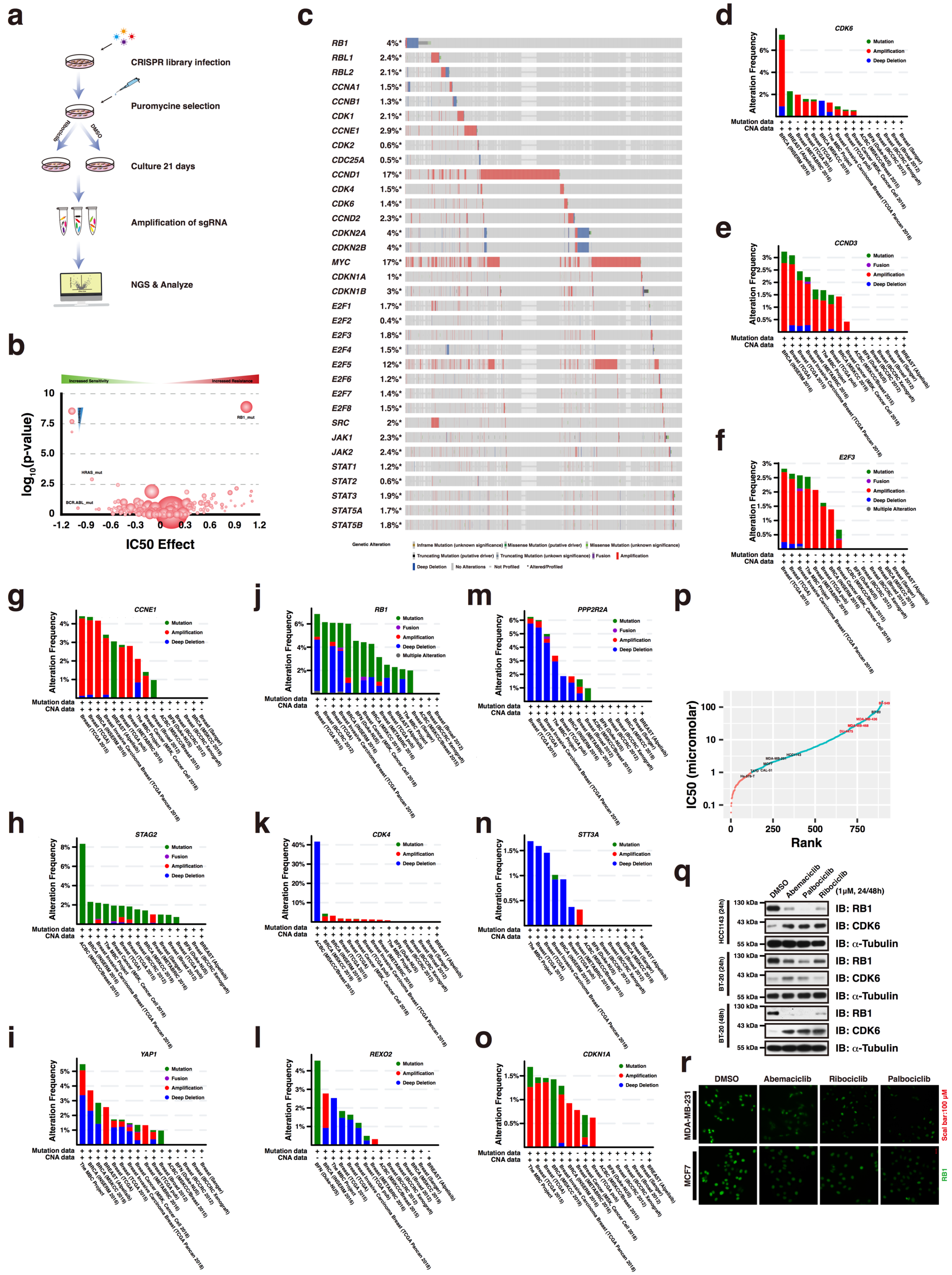

CRISPR library infection
Puromycine selection
Culture 21 days
Amplification of sgRNA
NGS & Analyze
IC50 Effect
log10(p-value)
RB1_mut
HRAS_mut
BCR.ABL_mut
Alteration Frequency
Mutation data
CNA data
CDK6
CCND3
E2F3
CCNE1
RB1
PPP2R2A
STAG2
CDK4
STT3A
YAP1
REXO2
CDKN1A
IC50 (micromolar)
Rank
IB: RB1
IB: CDK6
IB: α-Tubulin
DMSO
Abemaciclib
Palbociclib
Ribociclib
MDA-MB-231
MCF7

Supplementary Fig. 1. Genomic alterations in the cell cycle pathway in breast cancer.

(**a**) Workflow of the pooled CRISPR genetic screening strategy. (**b**) Genomic alteration and cellular response to palbociclib. Data was achieved from Genomics of Drug Sensitivity in Cancer database (https://www.cancerrxgene.org). (**c**) The type and frequency of genomic alterations in the cell cycle control genes in breast cancer. Data were obtained from the cBioPortal database. (**d-i**) Genomic mutation types and frequency of cell cycle genes whose amplification drive CDK4/6i resistance identified by the CRISPR screen. Data were obtained from the cBioPortal for Cancer Genomics database (https://www.cbioportal.org). (**j-o**) Genomic mutation types and frequency of cell cycle genes whose depletion drive CDK4/6i resistance identified by the CRISPR screen. Data were obtained from the cBioPortal database. (**p**) IC50 of cancer cell lines in response to palbociclib treatment. Data was achieved from Genomics of Drug Sensitivity in Cancer database (https://www.cancerrxgene.org). (**q**) Immunoblot showing the RB1 and CDK6 protein abundance in the context of CDK4/6i treatment in HCC1143 and BT-20 breast cancer cell lines. (r) Immunostaining showing decreased RB1 protein abundance in MCF7 and MDA-MB-231 breast cancer cells upon CDK4/6i treatment. Scale bar: 100 µM. The relevant raw data and uncropped blots are provided in Source Data.

Sup. Fig 2

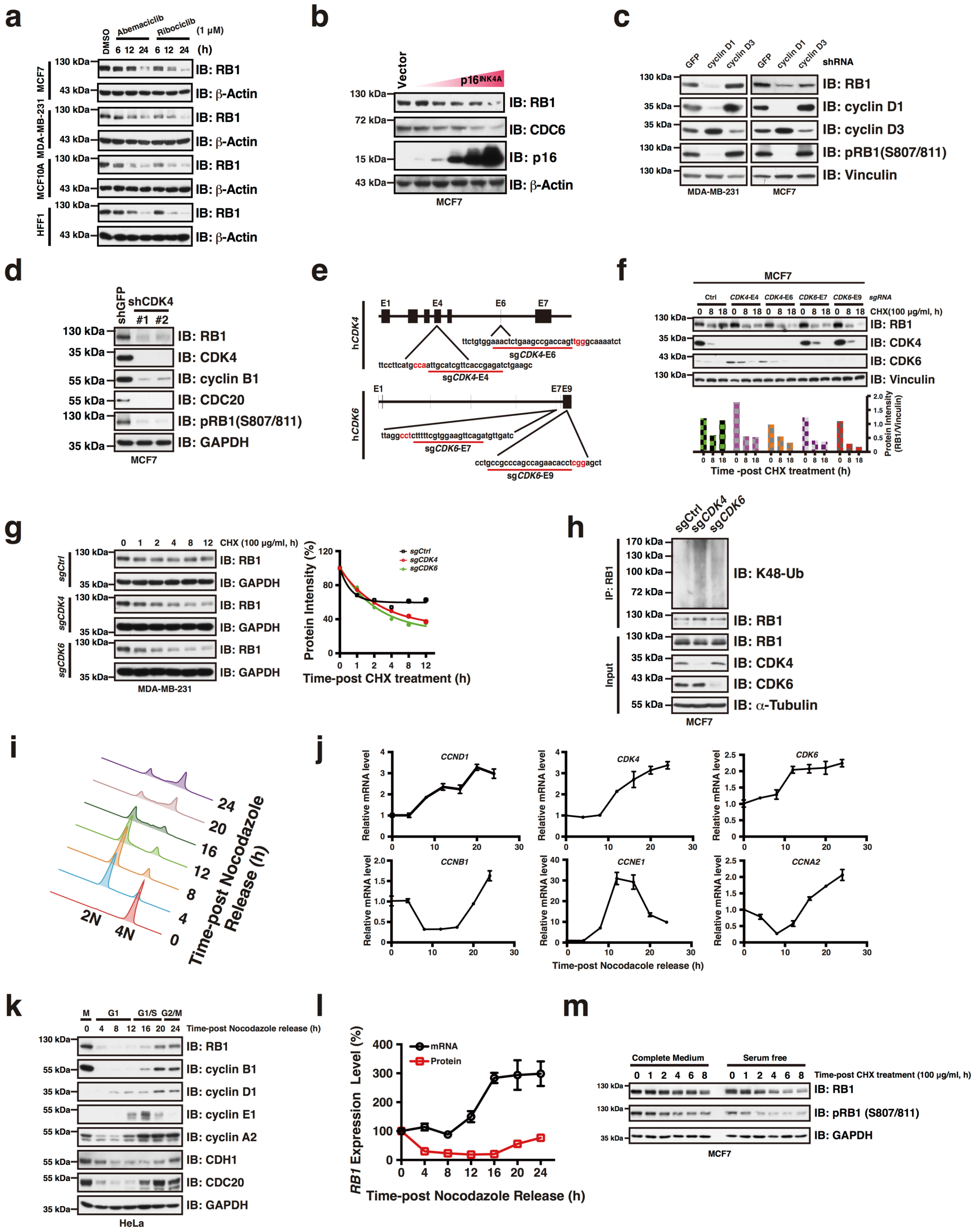
a
DMSO
Abemaciclib
Ribociclib
(1 μM)
6 12 24 6 12 24 (h)
MCF7
MDA-MB-231
MCF10A
HFF1
IB: RB1
IB: β-Actin
b
Vector
p16INK4A
IB: RB1
IB: CDC6
IB: p16
IB: β-Actin
MCF7
c
GFP cyclin D1 cyclin D3 GFP cyclin D1 cyclin D3 shRNA
IB: RB1
IB: cyclin D1
IB: cyclin D3
IB: pRB1(S807/811)
IB: Vinculin
MDA-MB-231
MCF7
d
shGFP
shCDK4
#1 #2
IB: RB1
IB: CDK4
IB: cyclin B1
IB: CDC20
IB: pRB1(S807/811)
IB: GAPDH
MCF7
e
hCDK4
ttctgtggaaactctgaagccgaccagttgggcaaaatct
sgCDK4-E6
ttccttcatgccaattgcatcgttcaccgagatctgaagc
sgCDK4-E4
hCDK6
ttaggcctctttttcgtggaagttcagatgttgatc
sgCDK6-E7
cctgccgcccagccagaacacctcggagct
sgCDK6-E9
f
MCF7
Ctrl CDK4-E4 CDK4-E6 CDK6-E7 CDK6-E9 sgRNA
CHX(100 μg/ml, h)
IB: RB1
IB: CDK4
IB: CDK6
IB: Vinculin
Protein Intensity (RB1/Vinculin)
Time -post CHX treatment (h)
g
CHX (100 μg/ml, h)
sgCtrl
sgCDK4
sgCDK6
IB: RB1
IB: GAPDH
MDA-MB-231
Protein Intensity (%)
Time-post CHX treatment (h)
h
sgCtrl sgCDK4 sgCDK6
IP: RB1
IB: K48-Ub
IB: RB1
Input
IB: RB1
IB: CDK4
IB: CDK6
IB: α-Tubulin
MCF7
i
2N
4N
Time-post Nocodazole Release (h)
j
CCND1
CDK4
CDK6
CCNB1
CCNE1
CCNA2
Relative mRNA level
Time-post Nocodacole release (h)
k
M G1 G1/S G2/M
Time-post Nocodazole release (h)
IB: RB1
IB: cyclin B1
IB: cyclin D1
IB: cyclin E1
IB: cyclin A2
IB: CDH1
IB: CDC20
IB: GAPDH
HeLa
l
mRNA
Protein
RB1 Expression Level (%)
Time-post Nocodazole Release (h)
m
Complete Medium
Serum free
Time-post CHX treatment (100 μg/ml, h)
IB: RB1
IB: pRB1 (S807/811)
IB: GAPDH
MCF7

Supplementary Fig. 2. Suppression of cyclin D1-CDK4 promotes RB1 degradation.

(**a**) Exposure of cells with CDK4/6i reduced RB1 protein abundance in a time-dependent manner in multiple breast cancer cell lines. **(b)** Ectopic expression of $p16^{INK4A}$ reduced RB1 protein levels in a dose-dependent manner in MCF7 cells. (**c**) Knocking-down of *cyclin D1* resulted in RB1 protein reduction in MCF7 and MDA-MB-231 breast cancer cells. (**d**) Knocking-down of *CDK4* led to RB1 protein reduction in MCF7 cells. (**e**) A schematic diagram of sgRNAs targeting the human *CDK4* and *CDK6* loci. (**f**) Knocking-out of *CDK4/6* accelerated RB1 protein degradation in MCF7 cells. (**g**) Knocking-out of *CDK4/6* accelerated RB1 degradation in MDA-MB-231 cells. (**h**) Knocking-out of *CDK4/6* resulted in accumulation of K48-linked polyubiquitination of RB1. (**i**) HeLa cells were treated with nocodazole (10 μM) for 16 hours and suspended cells were harvested and washed three times with PBS before seeding. Cells were then harvested at indicated time, and Fluorescence-activated cell sorting (FACS) analysis was performed to show cell distribution in different cell cycle phases. (**j**) RT-qPCR analysis showing the transcriptional pattern of cell cycle genes throughout the cell cycle progression (n=3). (**k**) Immunoblot showing that RB1 protein abundance fluctuates throughout the cell cycle. (**l**) Quantification of RB1 protein intensity and mRNA levels throughout the cell cycle (n=3). (**m**) CHX chase assay showing the destruction of RB1 was accelerated in the context of serum starvation in MCF7 cells. Data are presented as mean values +/- SEM. The relevant raw data and uncropped blots are provided in Source Data.

Sup. Fig 3

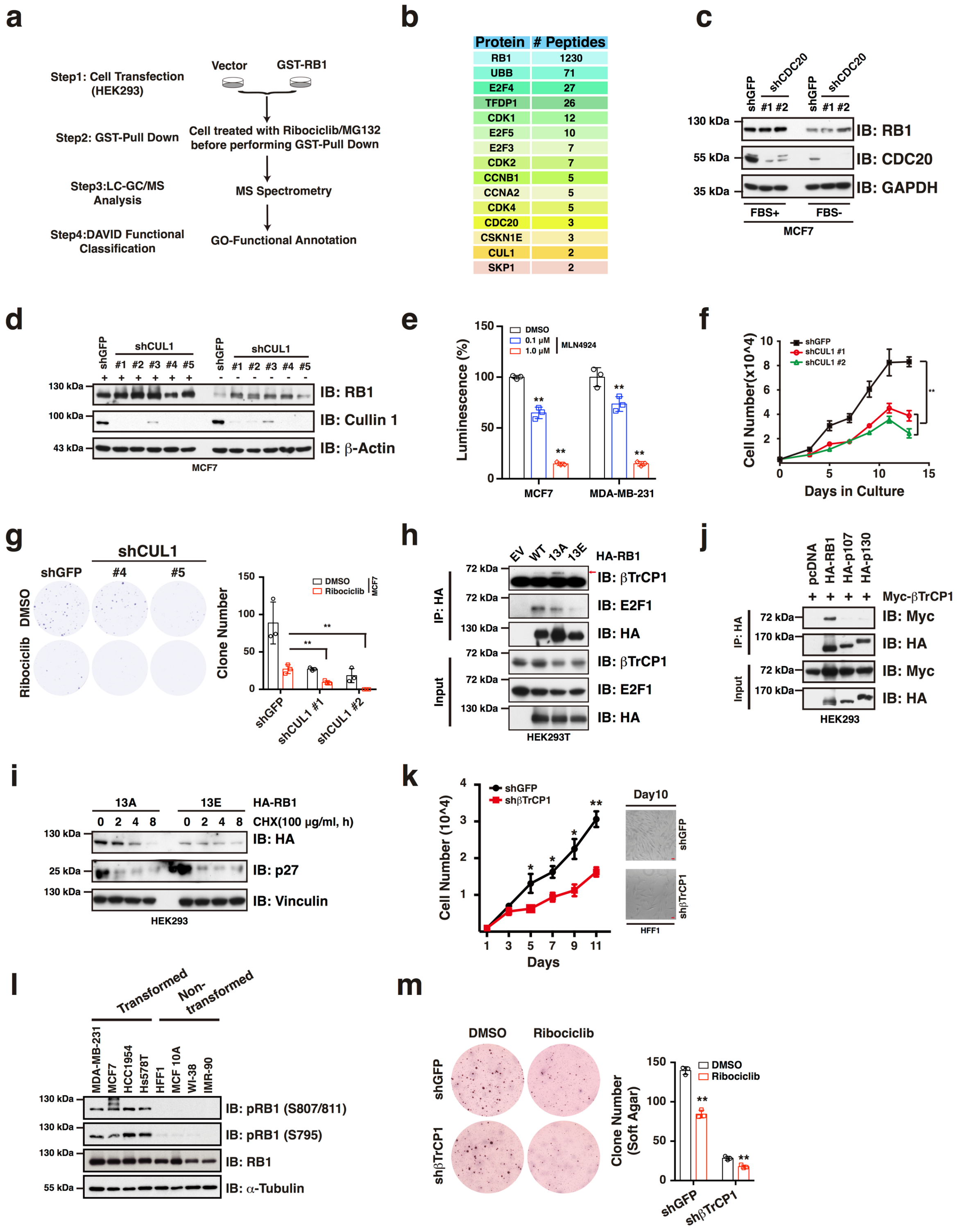
a
Step1: Cell Transfection (HEK293)
Vector
GST-RB1
Step2: GST-Pull Down
Cell treated with Ribociclib/MG132 before performing GST-Pull Down
Step3:LC-GC/MS Analysis
MS Spectrometry
Step4:DAVID Functional Classification
GO-Functional Annotation
b
Protein # Peptides
RB1 1230
UBB 71
E2F4 27
TFDP1 26
CDK1 12
E2F5 10
E2F3 7
CDK2 7
CCNB1 5
CCNA2 5
CDK4 5
CDC20 3
CSKN1E 3
CUL1 2
SKP1 2
c
shGFP
shCDC20
#1 #2
130 kDa
55 kDa
35 kDa
IB: RB1
IB: CDC20
IB: GAPDH
FBS+
FBS-
MCF7
d
shGFP
shCUL1
#1 #2 #3 #4 #5
130 kDa
100 kDa
43 kDa
IB: RB1
IB: Cullin 1
IB: β-Actin
MCF7
e
DMSO
0.1 μM
1.0 μM
MLN4924
Luminescence (%)
MCF7
MDA-MB-231
f
shGFP
shCUL1 #1
shCUL1 #2
Cell Number(x10^4)
Days in Culture
g
shCUL1
shGFP
#4
#5
DMSO
Ribociclib
Clone Number
MCF7
shGFP
shCUL1 #1
shCUL1 #2
h
EV WT 13A 13E
HA-RB1
IP: HA
Input
72 kDa
130 kDa
IB: βTrCP1
IB: E2F1
IB: HA
HEK293T
j
pcDNA
HA-RB1
HA-p107
HA-p130
Myc-βTrCP1
IP: HA
Input
72 kDa
170 kDa
IB: Myc
IB: HA
HEK293
i
13A
13E
HA-RB1
0 2 4 8
CHX(100 μg/ml, h)
130 kDa
25 kDa
IB: HA
IB: p27
IB: Vinculin
HEK293
k
shGFP
shβTrCP1
Cell Number (10^4)
Days
Day10
HFF1
l
Transformed
Non-transformed
MDA-MB-231
MCF7
HCC1954
Hs578T
HFF1
MCF 10A
WI-38
IMR-90
130 kDa
55 kDa
IB: pRB1 (S807/811)
IB: pRB1 (S795)
IB: RB1
IB: α-Tubulin
m
DMSO
Ribociclib
shGFP
shβTrCP1
Clone Number (Soft Agar)

Supplementary Fig. 3. Cullin 1-βTrCP1 mediates hypo-phosphorylated RB1 for proteasomal degradation in G1 phase.

(**a**) A schematic view of the workflow of LC-MS analysis. (**b**) A short list of proteins identified in GST-RB1 precipitates by LC-MS/MS. (**c**) Knocking-down of *CDC20* did not noticeably affect RB1 protein abundance in MCF7 cells. (**d)** Knocking-down of *Cullin 1* led to RB1 accumulation in the context of serum starvation. (**e**) Treatment of MCF7 and MDA-MB-231 cells with MLN4924 suppressed cell viability in a dose-dependent manner (n=3). The *P*-values were calculated by Student's t-test (two-sided). **: $P<0.01$. (**f**) Knocking-down of *Cullin 1* suppressed cell proliferation (n=4). Statistical differences were assessed by two-way ANOVA (two-sided). **: $P<0.01$. (**g**) Colony formation assay showing that knocking-down of *Cullin 1* enhanced the inhibitory effect of ribociclib on cell proliferation (n=3). The *P*-values were calculated by Student's t-test (two-sided). **: $P<0.01$. (**h)** Immunoprecipitation assay showing that the hyper-phosphorylation mimetic RB1-13E mutant was compromised in the RB1-βTrCP1 interaction. (**i**) CHX chase assay showing that the RB1-13E mutant was more stable than the RB1-13A mutant. (**j**) βTrCP1 specifically interacted with RB1, but not p107 and p130 in cells. (**k**) Knocking-down of endogenous *βTrCP1* suppressed HFF1 cell proliferation (n=4). Scale bar: 50 μM. The *P*-values were calculated by Student's t-test (two-sided). *: $P<0.05$, **: $P<0.01$. (**l**) Immunoblot showing that RB1 is hyper-phosphorylated in transformed cancer cells. (**m**) Soft agar assay showing that knocking-down of *βTrCP1* enhanced the inhibitory effect of ribociclib on MCF7 cell proliferation (n=3). The *P*-values were calculated by Student's t-test (two-sided). **: $P<0.01$. Data are presented as mean values +/- SEM. The relevant raw data and uncropped blots are provided in Source Data.

Sup. Fig 4

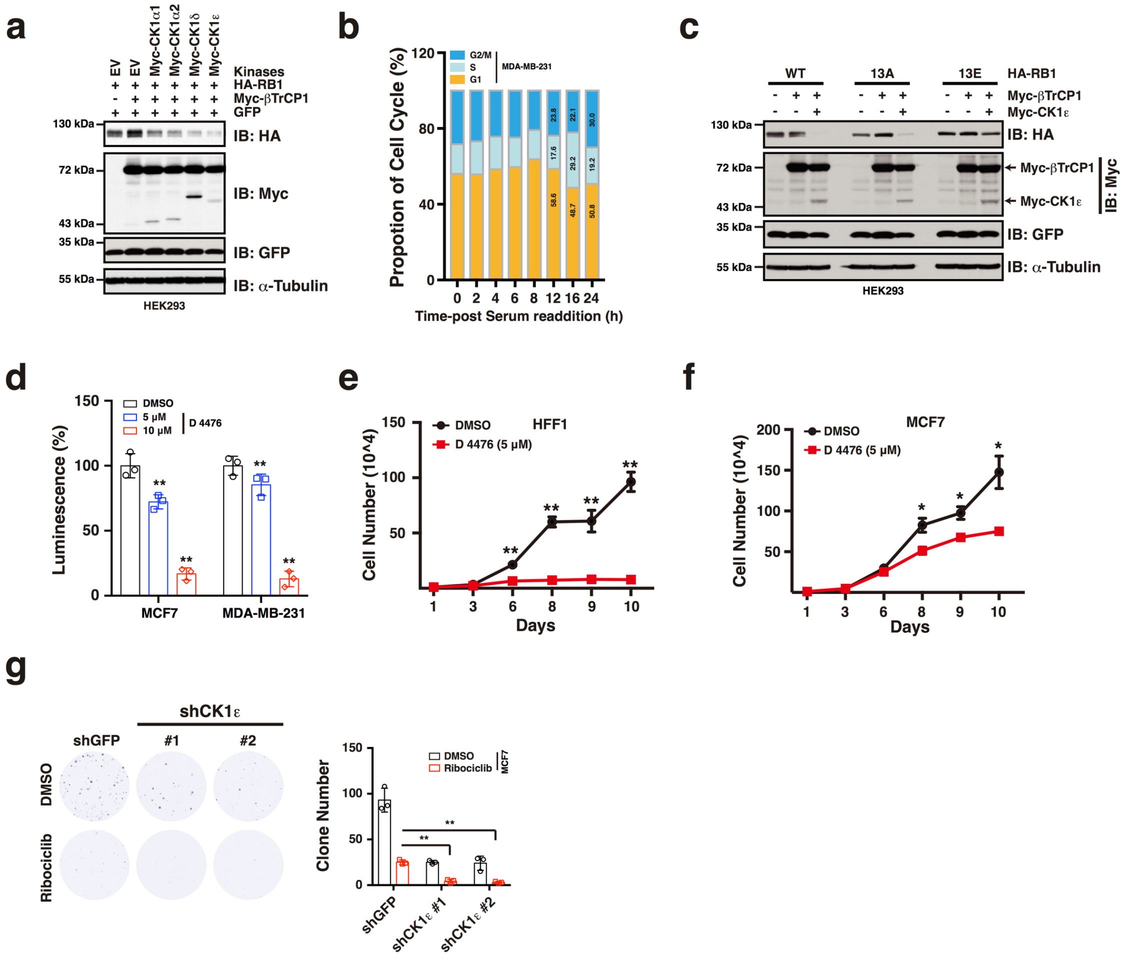
a
EV
EV
Myc-CK1α1
Myc-CK1α2
Myc-CK1δ
Myc-CK1ε
Kinases
HA-RB1
Myc-βTrCP1
GFP
130 kDa
72 kDa
43 kDa
35 kDa
55 kDa
IB: HA
IB: Myc
IB: GFP
IB: α-Tubulin
HEK293
b
G2/M
S
G1
MDA-MB-231
Propotion of Cell Cycle (%)
Time-post Serum readdition (h)
c
WT
13A
13E
HA-RB1
Myc-βTrCP1
Myc-CK1ε
IB: HA
Myc-βTrCP1
Myc-CK1ε
IB: Myc
IB: GFP
IB: α-Tubulin
HEK293
d
DMSO
5 μM
10 μM
D 4476
Luminescence (%)
MCF7
MDA-MB-231
e
DMSO
D 4476 (5 μM)
HFF1
Cell Number (10^4)
Days
f
DMSO
D 4476 (5 μM)
MCF7
Cell Number (10^4)
Days
g
shCK1ε
shGFP
#1
#2
DMSO
Ribociclib
Clone Number
MCF7
shGFP
shCK1ε #1
shCK1ε #2

Supplementary Fig. 4. CK1ε is required for βTrCP1-mediated RB1 degradation.

(**a**) Ectopic expression of CK1ε led to reduced RB1 protein abundance. (**b**) FACS analysis showing the distribution of MDA-MB-231 cells in different cycle phases at indicated time points post serum re-addition, related to Figure 3e. (**c**) Hyper-phosphorylation mimetic mutant RB1-13E was relatively resistant to CK1ε/βTrCP1-mediated degradation. (**d**) Exposure of cells with D 4476 reduced cell viability in a dose-dependent manner in MCF7 and MDA-MB-231 cells (n=3). The *P*-values were calculated by Student's t-test (two-sided). **: $P<0.01$. (**e**) Treatment of HFF1 non-transformed cells with D 4476 suppressed HFF1 cell proliferation (n=4). The *P*-values were calculated by Student's t-test (two-sided). **: $P<0.01$. (**f**) Treatment of MCF7 cells with D 4476 suppressed MCF7 cell proliferation (n=4). The *P*-values were calculated by Student's t-test (two-sided). **: $P<0.01$. (**g**) Colony formation assay showing that knocking-down of *CK1ε* enhanced the inhibitory effect of ribociclib on cell proliferation in MCF7 cells (n=3). The *P*-values were calculated by Student's t-test (two-sided). **: $P<0.01$. Data are presented as mean values +/- SEM. The relevant raw data and uncropped blots are provided in Source Data.

Sup. Fig 5

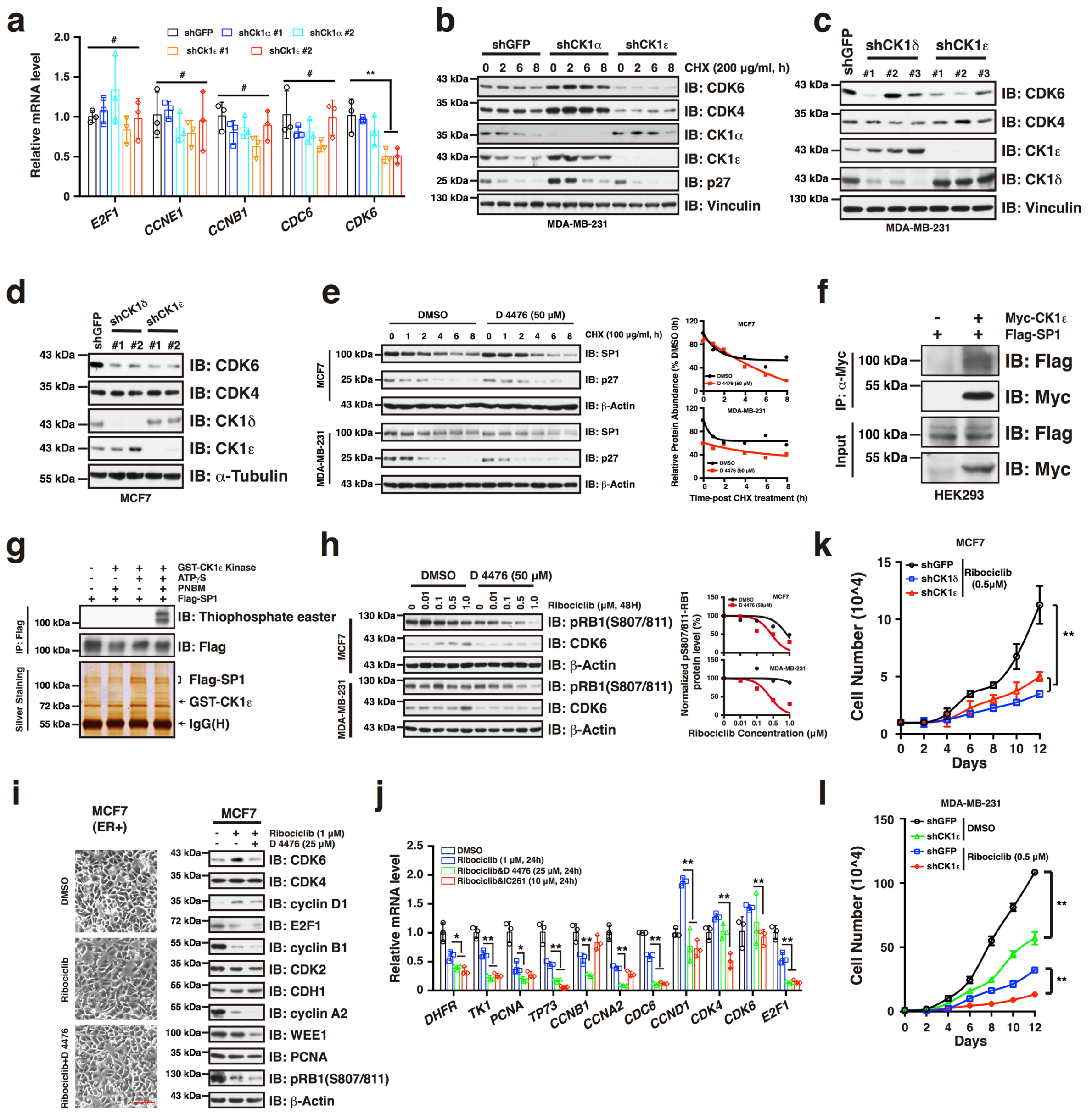

Supplementary Fig. 5. CK1ε-SP1 axis modulates *CDK6* upregulation in the context of CDK4/6i treatment.

(**a**) RT-qPCR analysis showing that knocking-down of *CK1ε* suppressed *CDK6* transcription (n=3). The *P*-values were calculated by Student's t-test (two-sided). #: no significant difference, **: *P*<0.01. (**b**) Knocking-down of *CK1ε* did not affect CDK6 protein stability. (**c**) Knocking-down of *CK1δ/ε* reduced CDK6 protein abundance in MDA-MB-231 cells. (**d**) Knocking-down of *CK1δ/ε* reduced CDK6 protein abundance in MCF7 cells. (**e**) Treatment of cells with D 4476 promoted SP1 degradation in MCF7 and MDA-MB-231 cells. (**f**) SP1 physically interacted with CK1ε in cells. (**g**) CK1ε phosphorylated SP1 *in vitro*. (**h**) Combination of D 4476 sensitized MCF7 and MDA-231 breast cancer cells to ribociclib treatment. (**i**) Combination of D 4476 with ribociclib prevented CDK6 accumulation and exhibited enhanced suppressive effect on cell cycle gene expression in MCF7 cells. (**j**) RT-qPCR analysis showing that combination of CDK4/6i (ribociclib) and CK1ε kinase inhibitor (D 4476 or IC261) abolished ribociclib-induced *CDK6* upregulation and exhibited enhanced inhibitory effect on cell cycle gene transcription in MDA-MB-231 cells (n=3). The *P*-values were calculated by Student's t-test (two-sided). *: *P*<0.05, **: *P*<0.01. (**k**) Knocking-down of *CK1ε* enhanced ribociclib-induced cell proliferation suppression in MCF7 cells (n=4). Statistical differences were assessed by two-way ANOVA (two-sided). **: *P*<0.01. (**l**) Knocking-down of CK1ε enhanced ribociclib-induced cell proliferation suppression in MDA-MB-231 cells (n=4). Statistical differences were assessed by two-way ANOVA (two-sided). **: *P*<0.01. Data are presented as mean values +/- SEM. The relevant raw data and uncropped blots are provided in Source Data.

Sup. Fig 6

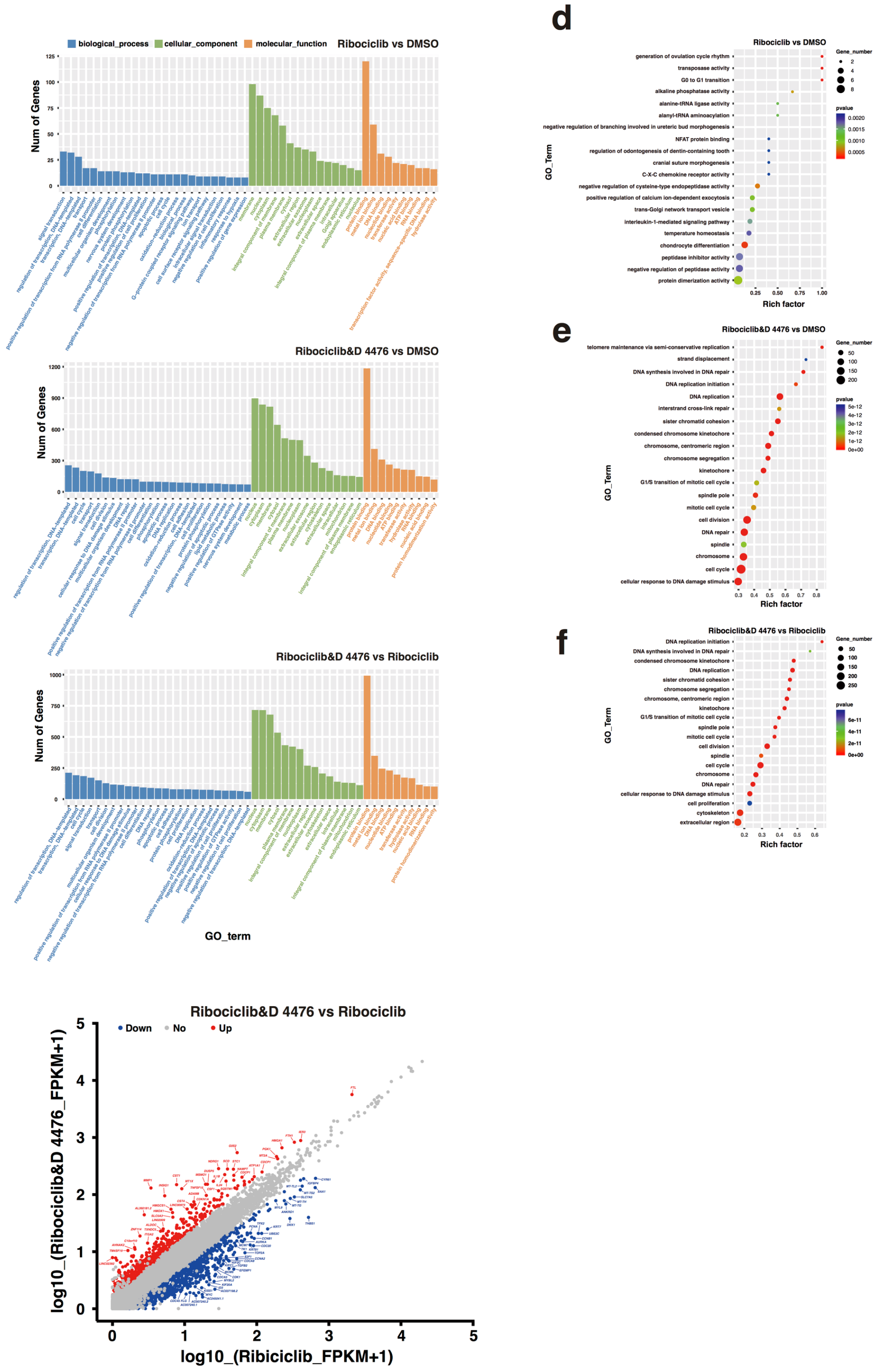
a
biological_process
cellular_component
molecular_function
Ribociclib vs DMSO
Num of Genes
b
Ribociclib&D 4476 vs DMSO
Num of Genes
c
Ribociclib&D 4476 vs Ribociclib
Num of Genes
GO_term
d
Ribociclib vs DMSO
generation of ovulation cycle rhythm
transposase activity
G0 to G1 transition
alkaline phosphatase activity
alanine-tRNA ligase activity
alanyl-tRNA aminoacylation
negative regulation of branching involved in ureteric bud morphogenesis
NFAT protein binding
regulation of odontogenesis of dentin-containing tooth
cranial suture morphogenesis
C-X-C chemokine receptor activity
negative regulation of cysteine-type endopeptidase activity
positive regulation of calcium ion-dependent exocytosis
trans-Golgi network transport vesicle
interleukin-1-mediated signaling pathway
temperature homeostasis
chondrocyte differentiation
peptidase inhibitor activity
negative regulation of peptidase activity
protein dimerization activity
GO_Term
Rich factor
Gene_number
pvalue
e
Ribociclib&D 4476 vs DMSO
telomere maintenance via semi-conservative replication
strand displacement
DNA synthesis involved in DNA repair
DNA replication initiation
DNA replication
interstrand cross-link repair
sister chromatid cohesion
condensed chromosome kinetochore
chromosome, centromeric region
chromosome segregation
kinetochore
G1/S transition of mitotic cell cycle
spindle pole
mitotic cell cycle
cell division
DNA repair
spindle
chromosome
cell cycle
cellular response to DNA damage stimulus
GO_Term
Rich factor
Gene_number
pvalue
f
Ribociclib&D 4476 vs Ribociclib
DNA replication initiation
DNA synthesis involved in DNA repair
condensed chromosome kinetochore
DNA replication
sister chromatid cohesion
chromosome segregation
chromosome, centromeric region
kinetochore
G1/S transition of mitotic cell cycle
spindle pole
mitotic cell cycle
cell division
spindle
cell cycle
chromosome
DNA repair
cellular response to DNA damage stimulus
cell proliferation
cytoskeleton
extracellular region
GO_Term
Rich factor
Gene_number
pvalue
g
Ribociclib&D 4476 vs Ribociclib
Down
No
Up
log10_(Ribociclib&D 4476_FPKM+1)
log10_(Ribiciclib_FPKM+1)

Supplementary Fig. 6. D 4476 synergizes with ribociclib in breast cancer cells.

MDA-MB-231 cells were treated with DMSO, ribociclib (1 μM) and ribociclib (1 μM) & D 4476 (25 μM) for 48 hours. Total RNAs were extracted and RNA-sequencing was performed. Gene expression profiles were then analyzed. Each treatment has three replicates. Cluster analysis showing the number of responsible genes involved in different cellular process: (**a**) ribociclib vs DMSO, (**b**) ribociclib & D 4476 vs DMSO, (**c**) ribociclib & D 4476 vs ribociclib. GO analysis showing the enriched cellular process upon drug treatment: (**d**) ribociclib vs DMSO, (**e**) ribociclib & D 4476 vs DMSO, (**f**) ribociclib & D 4476 vs ribociclib. (**g**) Scatter plot showing the up- and down-regulated genes in the ribociclib and D 4476 combination treatment compared to ribociclib single treatment.

Sup. Fig 7

a

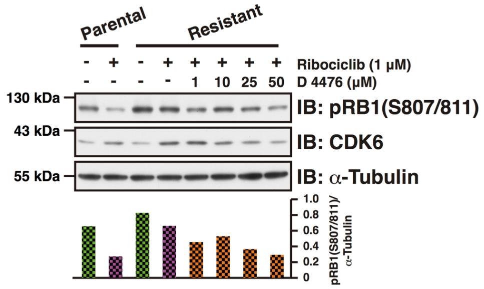

b

Parameter	Male	Female	Average	Unit
AUC(0-t)	3506.073	2642.218	3074.1±610.8	ug/L*h
MRT(0-t)	5.859	4.012	4.9±1.3	h
VRT(0-t)	88.971	52.692	70.8±25.7	h^2
t1/2z	11.473	4.682	8.1±4.8	h
Tmax	0.5	0.5	0.5	h
Vz/F	462.091	255.634	358.9±146.0	L/kg
CLz/F	27.913	37.834	32.9±7.0	L/h/kg
Cmax	2370.720	1938.420	2154.6±305.7	ug/L

c

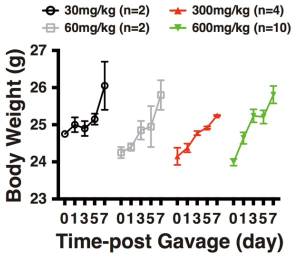

d

Male	spleen	liver	kidney	thymus	heart	testis	epididymis
Weight(g)	0.091±0.009	1.317±0.050	0.375±0.018	0.045±0.007	0.121±0.020	0.185±0.008	0.071±0.008
W-Range	0.07~0.12	1.0~1.4	0.3~0.4	0.03~0.08	0.09~0.15	0.16~0.22	0.05~0.08
Factor	0.355±0.028	5.156±0.128	1.467±0.051	0.177±0.021	0.473±0.074	0.726±0.037	0.280±0.035
F-Range	0.30~0.41	4.1~5.5	0.8~1.6	0.11~0.25	0.30~0.58	0.50~0.78	0.15~0.30
Female	spleen	liver	kidney	thymus	heart	ovary	uterus
Weight(g)	0.107±0.014	0.863±0.072	0.252±0.021	0.047±0.005	0.092±0.004	0.016±0.003	0.166±0.062
W-Range	0.07~0.12	0.7~1.2	0.2~0.3	0.03~0.07	0.08~0.14	0.01~0.03	0.09~0.32
Factor	0.411±0.059	3.311±0.320	0.968±0.087	0.182±0.024	0.353±0.017	0.062±0.012	0.639±0.251
F-Range	0.33~0.50	2.7~3.9	0.9~1.4	0.11~0.25	0.25~0.50	0.04~0.09	0.40~1.15

e 4T1 Xenograft

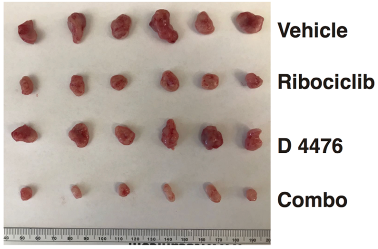

f MDA-MB-231 Xenograft

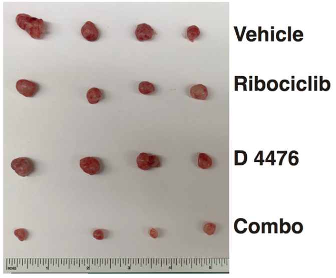

g PDX Model

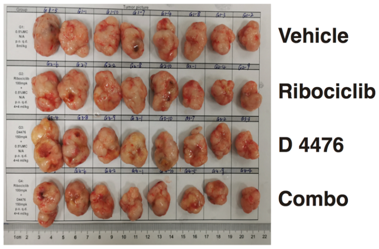

h

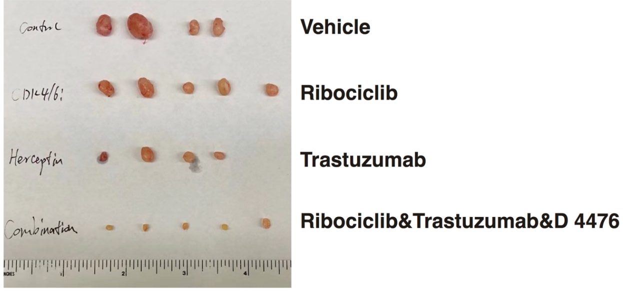

Supplementary Fig. 7. Pharmacokinetic properties of D 4476 *in vivo*.

(**a**) Combination of D 4476 and ribociclib abolished CDK6 upregulation and overcomes CDK4/6i resistance on inhibiting RB1 phosphorylation in MDA-MB-231 cells. (**b**) A table showing the major pharmacokinetic parameters of D 4476 *in vivo*. (**c**) Administration of D4476 did not noticeably affect the gain of body weight. Data are presented as mean values +/- SEM. (**d**) Administration of D4476 had no noticeable effect on organ weight. (**e-h**) 4T1 xenograft tumors (**e**), MDA-MB-231 xenograft tumors (**f**), PDX tumors (**g**) and BT-474 xenograft tumors (**h**), treated with indicated therapeutic strategy. The relevant raw data and uncropped blots are provided in Source Data.

**Supplementary Table 1. Primers for gene cloning**	
Name	Sequence
sgControl	CGCTTCCGCGGCCCGTTCAA
sgRNA-*hCDK4*-F #1	CACCGATCTCGGTGAACGATGCAAT
sgRNA-*hCDK4*-R #1	AAACATTGCATCGTTCACCGAGATC
sgRNA-*hCDK4*-F #2	CACCGAAACTCTGAAGCCGACCAGT
sgRNA-*hCDK4*-R #2	AAACACTGGTCGGCTTCAGAGTTTC
sgRNA-*hCDK6*-F #1	CACCGTCTGAACTTCCACGAAAAAG
sgRNA-*hCDK6*-R #1	AAACCTTTTTCGTGGAAGTTCAGAC
sgRNA-*hCDK6*-F #2	CACCGCCGCCCAGCCAGAACACCT
sgRNA-*hCDK6*-R #2	AAACAGGTGTTCTGGCTGGGCGGC
hCDK6-p1000-F	GCATGGTACCGCTCCTCTGAAATACGTTAGTGAACC
hCDK6-p800-F	GCATGGTACCCAAAACAAATGTAGCTCATGCTG
hCDK6-p600-F	GCATGGTACCGTGTTTTTCCCATCCGTCCAC
hCDK6-p300-F	GCATGGTACCCGCCTCATTTCTCTCCGGAAGGC
hCDK6-p100-F	GCATGGTACCGCGAGCGGCGCGGGCGGCGCC
hCDK6-promoter-R	GCATCTCGAGAGACTCTGGGGAAGGAGTTACCAGCAC
hCDK6-dGC box-F	CGCCCTCCGCCGTCCCTCCGCCC
hCDK6-dGC box-R	GCGCCGCTCGCTGGGGGCGGACG
hRB1-GST-F401	GCATGGATCCCCACACACTCCAGTTAGGAC
hRB1-GST-R401	GCATCTCGAGCTACTGCAAAATATTTGTTTTCAG
hRB1-S508A/T510A-F	CAGAATCTTGATGCCGGAGCCGATTTGTCTTTCCC
hRB1-S508A/T510A-R	GGGAAAGACAAATCGGCTCCGGCATCAAGATTCTG
hRB1-S508A/S513A-F	CAGAATCTTGATGCCGGAACAGATTTGGCCTTCCCATG
hRB1-S508A/S513A-R	CATGGGAAGGCCAAATCTGTTCCGGCATCAAGATTCTG
hRB1-S508/T510/S513A-F	CAGAATCTTGATGCCGGAGCCGATTTGGCCTTCCCATG
hRB1-S508/T510/S513A-R	CATGGGAAGGCCAAATCGGCTCCGGCATCAAGATTCTG

**Supplementary Table 2. Real-Time PCR primers**	
Name	Sequence
*hRB1*_Forward	TTGGATCACAGCGATACAAACTT
*hRB1*_Reverse	AGCGCACGCCAATAAAGACAT
*hE2F1*_Forward	ACGTGACGTGTCAGGACCT
*hE2F1*_Reverse	GATCGGGCCTTGTTTGCTCTT
*hCCNA2*_Forward	CGCTGGCGGTACTGAAGTC
*hCCNA2*_Reverse	GAGGAACGGTGACATGCTCAT
*hCCNE1*_Forward	GCCAGCCTTGGGACAATAATG
*hCCNE1*_Reverse	CTTGCACGTTGAGTTTGGGT
*hDHFR*_Forward	GCCACCGCTCAGGAATGAAT
*hDHFR*_Reverse	GAGCTCCTTGTGGAGGTTCC
*hTK1*_Forward	GCCAAAGACACTCGCTACAG
*hTK1*_Reverse	CCCCTCGTCGATGCCTATG
*hPCNA*_Forward	CCTGCTGGGATATTAGCTCCA
*hPCNA*_Reverse	CAGCGGTAGGTGTCGAAGC
*hCDC6*_Forward	ACCTATGCAACACTCCCCATT
*hCDC6*_Reverse	TGGCTAGTTCTCTTTTGCTAGGA
*hTP73*_Forward	GACGAGGACACGTACTACCTT
*hTP73*_Reverse	CTGCCGATAGGAGTCCACCA
*hCCND1*_Forward	GCTGCGAAGTGGAAACCATC
*hCCND1*_Reverse	CCTCCTTCTGCACACATTTGAA
*hCDK4*_Forward	ATGGCTACCTCTCGATATGAGC
*hCDK4*_Reverse	CATTGGGGACTCTCACACTCT
*hCDK6*_Forward	TCTTCATTCACACCGAGTAGTGC
*hCDK6*_Reverse	TGAGGTTAGAGCCATCTGGAAA
*hCCNB1*_Forward	AATAAGGCGAAGATCAACATGGC
*hCCNB1*_Reverse	TTTGTTACCAATGTCCCCAAGAG
*hACTN1*_Forward	GCTCGTCGTCGACAACGGCTC
*hACTN1*_Reverse	CAAACATGATCTGGGTCATCTTCTC
hCDK6-ChIP-F	CTCATTTCTCTCCGGAAGGC
hCDK6-ChIP-R	GCAGCTGGGGCGACCGTGCG
