# Supplementary material for: Inhibition of CK1ε potentiates the therapeutic efficacy of CDK4/6 inhibitor in breast cancer
Source: Nat Commun. 2021 Sep 10;12:5386. doi: 10.1038/s41467-021-25700-6 (PMC8433397; doi:10.1038/s41467-021-25700-6)
Supplement: Supplementary file 2 — Description of Additional Supplementary Files [file 41467_2021_25700_MOESM2_ESM.docx]

**Description of Additional Supplementary Files**

File Name: Supplementary Data 1

Description: CRISPR/Cas9-mediated genetic screen results in MCF7 and MDA-MB-231 breast cancer cells

File Name: Supplementary Data 2

Description: LC-MS results showing the proteins identified in GST-RB1 pull-down precipitates

|  |
| --- |

|  |
| --- |
