# Supplementary material for: Inhibition of CK1ε potentiates the therapeutic efficacy of CDK4/6 inhibitor in breast cancer
Source: Nat Commun. 2021 Sep 10;12:5386. doi: 10.1038/s41467-021-25700-6 (PMC8433397; doi:10.1038/s41467-021-25700-6)
Supplement: Supplementary file 5 — Reporting Summary [file 41467_2021_25700_MOESM5_ESM.pdf]

nature portfolio

Corresponding author(s): Wenyi Wei

Last updated by author(s): Aug 9, 2021

Reporting Summary

Nature Portfolio wishes to improve the reproducibility of the work that we publish. This form provides structure for consistency and transparency in reporting. For further information on Nature Portfolio policies, see our Editorial Policies and the Editorial Policy Checklist.

Statistics

For all statistical analyses, confirm that the following items are present in the figure legend, table legend, main text, or Methods section.

n/a	Confirmed	
☐	☒	The exact sample size (*n*) for each experimental group/condition, given as a discrete number and unit of measurement
☐	☒	A statement on whether measurements were taken from distinct samples or whether the same sample was measured repeatedly
☐	☒	The statistical test(s) used AND whether they are one- or two-sided<br>*Only common tests should be described solely by name; describe more complex techniques in the Methods section.*
☒	☐	A description of all covariates tested
☒	☐	A description of any assumptions or corrections, such as tests of normality and adjustment for multiple comparisons
☐	☒	A full description of the statistical parameters including central tendency (e.g. means) or other basic estimates (e.g. regression coefficient) AND variation (e.g. standard deviation) or associated estimates of uncertainty (e.g. confidence intervals)
☐	☒	For null hypothesis testing, the test statistic (e.g. *F*, *t*, *r*) with confidence intervals, effect sizes, degrees of freedom and *P* value noted<br>*Give P values as exact values whenever suitable.*
☒	☐	For Bayesian analysis, information on the choice of priors and Markov chain Monte Carlo settings
☒	☐	For hierarchical and complex designs, identification of the appropriate level for tests and full reporting of outcomes
☒	☐	Estimates of effect sizes (e.g. Cohen's *d*, Pearson's *r*), indicating how they were calculated

Our web collection on statistics for biologists contains articles on many of the points above.

Software and code

Policy information about availability of computer code

Data collection	N/A
Data analysis	ImageJ (1.48v), FlowJo (V10.4.0), Microsoft Excel for Mac (v15.36), GraphPad Prism (v7a), GSEA (v4.1.0), Bowtie (2v2.2.9), MACS2 (v2.1.1), GREAT (v4.0.4), R package-edgeR (v3.34.0) and CytExpert (v2.3), HISAT2 (2.0.4), StringTie (1.3.4), Ballgown (2.24.0), edgeR (v3.34.0).

For manuscripts utilizing custom algorithms or software that are central to the research but not yet described in published literature, software must be made available to editors and reviewers. We strongly encourage code deposition in a community repository (e.g. GitHub). See the Nature Portfolio guidelines for submitting code & software for further information.

Data

Policy information about availability of data

All manuscripts must include a data availability statement. This statement should provide the following information, where applicable:

- Accession codes, unique identifiers, or web links for publicly available datasets
- A description of any restrictions on data availability
- For clinical datasets or third party data, please ensure that the statement adheres to our policy

The following Data availability section has been included in the Methods section: The RNA sequencing data generated in this study have been deposited in the Gene Expression Omnibus Database under accession code GSE177054 (https://www.ncbi.nlm.nih.gov/geo/query/acc.cgi?acc=GSE177054). The CRISPR screen data generated in this study are provided in Supplementary Data 1. The MS data generated in this study are provided in Supplementary Data 2. The human cancer data were derived from cBioPortal for cancer genomics (https://www.cbioportal.org). The data for Supplementary Figure 1b and 1p were achieved from Genomics of Drug Sensitivity in Cancer database (https://www.cancerrxgene.org). Publicly available E2F1 and SP1 ChIP-seq datasets from MCF7 cells (Fig. 4g) were obtained from the GEO database (GSE92014: https://www.ncbi.nlm.nih.gov/geo/query/acc.cgi and GSE31477: https://www.ncbi.nlm.nih.gov/geo/query/acc.cgi). These datasets

March 2021

were aligned to the hg38 genome using the Bowtie 2v2.2.9. Peak calling was performed using MACS2 v2.1.1 with default parameters except '-q 0.001'. The GREAT software v4.0.4 (http://great.stanford.edu/public/html/) was used to assign peaks to genes (within 500 bp up- or downstream of the TSS). DAVID bioinformatics Database v6.8 can be accessed through .https://david.ncifcrf.gov/tools.jsp). Source data are provided with this paper.

Field-specific reporting

Please select the one below that is the best fit for your research. If you are not sure, read the appropriate sections before making your selection.

☒ Life sciences ☐ Behavioural & social sciences ☐ Ecological, evolutionary & environmental sciences

For a reference copy of the document with all sections, see nature.com/documents/nr-reporting-summary-flat.pdf

Life sciences study design

All studies must disclose on these points even when the disclosure is negative.

Sample size	No statistical method was used to calculate sample size. Sample size was determined to be adequate based on the magnitude and consistency of measurable differences between groups. The size of animal studies was included in the figure legends. Sample sizes were chosen as large as possible while taking into account the experimental effort required to generate the respective data
Data exclusions	No data was excluded from the experiments.
Replication	All experimental findings were reliably reproduced. Multiple independent repeats were included for related experiments. Each experiment was performed for at least twice to make sure similar results are reproducible. Animal-related xenograft studies have been done once for each breast cancer cell line but repeated using different xenograft models.
Randomization	For all in vivo experiments, animals were randomly assigned into different treatment groups after tumor inoculation. The starting tumor burden in the treatment and control groups was similar before treatment. For experiments other than in vivo studies, samples were randomly assigned into experimental groups.
Blinding	The investigators were not blinded to group allocation during data collection and/or analysis because we measured the value of tumor size and gave different treatments among groups.

Reporting for specific materials, systems and methods

We require information from authors about some types of materials, experimental systems and methods used in many studies. Here, indicate whether each material, system or method listed is relevant to your study. If you are not sure if a list item applies to your research, read the appropriate section before selecting a response.

Materials & experimental systems

n/a	Involved in the study	
☐	☒	Antibodies
☐	☒	Eukaryotic cell lines
☒	☐	Palaeontology and archaeology
☐	☒	Animals and other organisms
☒	☐	Human research participants
☒	☐	Clinical data
☒	☐	Dual use research of concern

Methods

n/a	Involved in the study	
☒	☐	ChIP-seq
☐	☒	Flow cytometry
☒	☐	MRI-based neuroimaging

Antibodies

Antibodies used	Antibodies used in this study are as follows: anti-RB1 (#9309), anti-CDK2 (#2546), anti-CDK4 (#12790), anti-CDK6 (#3136), anticyclin D1 (#2978), anti-cyclin E1 (#4129), anti-SKP2 (#4358), anti-WEE1 (#4936), anti-BRAF (#9433), anti-Cullin 1(#4495), antipRB1(S807/811) (#9308), anti-AKT2 (#3063), anti-SP1 (#9389), anti-GAPDH (#2118), anti-cyclin A2 (#4656), anti-TrCP1 (#4394), anti-K48-Ubquitin (#4289), anti-CDC6 (#3387), anti-Myc (#2278), anti-GST (#2625), anti-HER2 (#2242), anti-Lamin A/C (#4777), anti-MEK (#9122), anti-PCNA (#2586), anti-p16 (#80772) and anti-GFP (#2555) were purchased from Cell Signaling Technology. Anti-cyclin D3 (sc-6283), anti-XPO7 (sc-390025), anti-PTEN (sc-7974), anti-CDH1 (sc-56312), anti-E2F1 (sc-251), anti-cyclin B1 (sc-7393), anti-CDC20 (sc-13162), anti-CK1sc-, anti-CK1(sc-6471), anti-CK1(sc-6474), anti-- Tubulin (sc-5286), anti--Actin (sc-47778), anti-p53 (sc-126) and anti-BrdU (sc-32323) were purchased from Santa Cruz. Anti-HA (H6908), anti-Flag (F3165) and anti-Vinculin (V9131) were purchased form Sigma. Anti-p27 (610242) was purchased from BD Biosciences. Anti-Thiophosphate ester antibody (ab133473) and anti-FBW7 (ab109617) were purchased from abcam. Alexa fluor® 488 (Cat #710369) was purchased from Invitrogen.

March 2021

Validation

anti-RB1 antibody used for immunoblotting was validated by shRNA-based knockdown in MCF7 cells.
anti-CDK6 antibody used for immunoblotting was validated by CRISPR/Cas9-mediated knockout in MCF7 and MDA-MB-231 cells.
anti-CDK4 antibody used for immunoblotting was validated by CRISPR/Cas9-mediated knockout in MCF7 and MDA-MB-231 cells.
anti-CK1a, CK1e and CK1d antibody used for immunoblotting was validated by CRISPR/Cas9-mediated knockout in MDA-MB-231 cells.
anti-CDC20 antibody used for immunoblotting was validated by shRNA-based knockdown in MCF7 cells.
anti-Cullin1 antibody used for immunoblotting was validated by shRNA-based knockdown in MCF7 cells.
anti-bTrCP1 antibody used for immunoblotting was validated by shRNA-based knockdown in MCF7 and MDA-MB-231 cells.
anti-CDH1 antibody used for immunoblotting was validated by shRNA-based knockdown in MCF7 and MDA-MB-231 cells.
anti-cyclin D1 and D3 antibody used for immunoblotting was validated by shRNA-based knockdown in MCF7 and MDA-MB-231 cells.

anti-CDK2 antibody: https://www.cellsignal.com/products/primary-antibodies/cdk2-78b2-rabbit-mab/2546.

anti-cyclin E1 antibody: https://www.cellsignal.com/products/primary-antibodies/cyclin-e1-he12-mouse-mab/4129?site-search-type=Products&N=4294956287&Ntt=%234129&fromPage=plp&_requestid=2668756.

anti-SKP2 antibody: https://www.cellsignal.com/products/primary-antibodies/skp2-antibody/4358?site-search-type=Products&N=4294956287&Ntt=%234358&fromPage=plp&_requestid=2668796.

anti-WEE1 antibody: https://www.cellsignal.com/products/primary-antibodies/wee1-antibody/4936.

anti-BRAF antibody: https://www.cellsignal.com/products/primary-antibodies/b-raf-55c6-rabbit-mab/9433?site-search-type=Products&N=4294956287&Ntt=%239433&fromPage=plp&_requestid=2668950.

anti-pRB1 (S807/811) antibody: https://www.cellsignal.com/products/primary-antibodies/phospho-rb-ser807-811-antibody/9308?site-search-type=Products&N=4294956287&Ntt=%239308&fromPage=plp&_requestid=2669034.

anti-AKT2 antibody: https://www.cellsignal.com/products/primary-antibodies/akt2-d6g4-rabbit-mab/3063.

anti-SP1 antibody: https://www.cellsignal.com/products/primary-antibodies/sp1-d4c3-rabbit-mab/9389?site-search-type=Products&N=4294956287&Ntt=%239389&fromPage=plp&_requestid=2669105.

anti-GAPDH antibody: https://www.cellsignal.com/products/primary-antibodies/gapdh-14c10-rabbit-mab/2118.

anti-cyclin A2 antibody: https://www.cellsignal.com/products/primary-antibodies/cyclin-a2-bf683-mouse-mab/4656?site-search-type=Products&N=4294956287&Ntt=%234656&fromPage=plp&_requestid=2669200.

anti-K48-Ubiquitin antibody: https://www.cellsignal.com/products/primary-antibodies/k48-linkage-specific-polyubiquitin-antibody/4289?site-search-type=Products&N=4294956287&Ntt=%234289&fromPage=plp&_requestid=2669250.

anti-CDC6 antibody: https://www.cellsignal.com/products/primary-antibodies/cdc6-c42f7-rabbit-mab/3387?site-search-type=Products&N=4294956287&Ntt=%233387&fromPage=plp&_requestid=2669304.

anti-Myc-Tag antibody: https://www.cellsignal.com/products/primary-antibodies/myc-tag-71d10-rabbit-mab/2278?site-search-type=Products&N=4294956287&Ntt=%232278&fromPage=plp.

anti-GST antibody: https://www.cellsignal.com/products/primary-antibodies/gst-91g1-rabbit-mab/2625?site-search-type=Products&N=4294956287&Ntt=%232625&fromPage=plp&_requestid=2669405.

anti-HER2 antibody: https://www.cellsignal.com/products/primary-antibodies/her2-erbb2-antibody/2242?site-search-type=Products&N=4294956287&Ntt=%232242&fromPage=plp&_requestid=2669441.

anti-Lamin A/C antibody: https://www.cellsignal.com/products/primary-antibodies/lamin-a-c-4c11-mouse-mab/4777?site-search-type=Products&N=4294956287&Ntt=%234777&fromPage=plp&_requestid=2669475.

anti-MEK1/2 antibody: https://www.cellsignal.com/products/primary-antibodies/mek1-2-antibody/9122?site-search-type=Products&N=4294956287&Ntt=%239122&fromPage=plp&_requestid=2669511.

anti-PCNA antibody: https://www.cellsignal.com/products/primary-antibodies/pcna-pc10-mouse-mab/2586?site-search-type=Products&N=4294956287&Ntt=%232586&fromPage=plp&_requestid=2669549.

anti-p16 antibody: https://www.cellsignal.com/products/primary-antibodies/p16-ink4a-d7c1m-rabbit-mab/80772?site-search-type=Products&N=4294956287&Ntt=%2380772&fromPage=plp&_requestid=2669586.

anti-GFP antibody: https://www.cellsignal.com/products/primary-antibodies/gfp-antibody/2555?site-search-type=Products&N=4294956287&Ntt=%232555&fromPage=plp&_requestid=2669620.

anti-a-Tubulin antibody: https://www.cellsignal.com/products/primary-antibodies/a-tubulin-dm1a-mouse-mab/3873?site-search-type=Products&N=4294956287&Ntt=tubulin&fromPage=plp.

Anti-b-Actin antibody: https://www.cellsignal.com/products/primary-antibodies/b-actin-8h10d10-mouse-mab/3700?site-search-

type=Products&N=4294956287&Ntt=actin&fromPage=plp.

anti-XPO7 antibody: https://www.scbt.com/p/exportin-7-antibody-a-11?productCanUrl=exportin-7-antibody-a-11&_requestid=4043181.

anti-PTEN antibody: https://www.scbt.com/p/pten-antibody-a2b1?requestFrom=search.

Anti-E2F1 antibody: https://www.scbt.com/p/e2f-1-antibody-kh95?requestFrom=search.

anti-cyclin B1 antibody: https://www.scbt.com/p/cyclin-b1-antibody-d-11?requestFrom=search.

Anti-p53 antibody: https://www.scbt.com/p/p53-antibody-do-1?requestFrom=search.

anti-BrdU antibody: https://www.scbt.com/p/brdu-antibody-iib5?requestFrom=search.

anti-HA antibody: https://www.sigmaaldrich.com/US/en/product/SIGMA/H6908.

anti-Flag antibody: https://www.sigmaaldrich.com/US/en/product/sigma/f3165?context=product.

anti-Vinculin antibody: https://www.sigmaaldrich.com/US/en/product/sigma/v9131?context=product.

anti-p27 antibody: https://www.bdbiosciences.com/en-us/products/reagents/microscopy-imaging-reagents/immunofluorescence-reagents/purified-mouse-anti-p27-kip1.610242.

anti-Thiophosphate easter antibody: https://www.abcam.com/thiophosphate-ester-antibody-51-8-ab133473.html.

anti-FBXW7 antibody: https://www.abcam.com/fbxw7-antibody-ab109617.html.

anti-Alexa fluor 488 polyclonal antibody: https://www.thermofisher.com/antibody/product/Alexa-Fluor-488-Antibody-Polyclonal/A-11094.

Eukaryotic cell lines

Policy information about cell lines

Cell line source(s)	MCF7, MDA-MB-231, SK-BR-3, HFF1, MCF10A, HCC1954, Hs587T, WI-38, IMR-90, BT-474, MDA-MB-453, HCC1143, CAL51, BT-549, Hs587T, BT-20, HEK293T, HEK293, and HeLa were purchased from ATCC.
Authentication	All cell lines used in this study were authenticated by STR profile report.
Mycoplasma contamination	All cell lines in our laboratory were routinely tested for mycoplasma contamination and cells used in this study were mycoplasma free.
Commonly misidentified lines (See ICLAC register)	Among all the cell lines, only BT-20 was listed by ICLAC in the Table 2, indicates that“cell lines where some stocks have been shown to be misidentified, but where authentic stock is known to exist”.We have shown the ATCC authentication report (STR method, 100% match to ATCC cell line HTB-19 (BT-20)) in our previous work (Gao Y. et al, 2020, Nat Cell Biol, doi: 10.1038/s41556-020-0562-4). The reason we use BT-20 in our study is because this cell line is less sensitive to CDK4/6 inhibitor treatment than MCF7 and MDA-MB-231. We want to test if our finding also works in CDK4/6 inihibitor less sensitive cell lines.

Animals and other organisms

Policy information about studies involving animals; ARRIVE guidelines recommended for reporting animal research

Laboratory animals	Female 6- to 8-week-old BALB/c or BALB/c nude mice, Female and male 6- to 8-week-old ICR mice.
Wild animals	The study did not involve wild animals.
Field-collected samples	The study did not involve samples collected from field.
Ethics oversight	Animal studies were approved by Dana-Farber Cancer Institute Institutional Animal Care and Use Committee (IACUC; protocol number 11-009) and by Beth Israel DeaconessMedical Center Institutional Animal Care and Use Committee (IACUC; protocol number 043-2015), and performed in accordance with guidelines established by NIH Guide for the care and use of laboratory animals.

Note that full information on the approval of the study protocol must also be provided in the manuscript.

Flow Cytometry

Plots

Confirm that:

- [x] The axis labels state the marker and fluorochrome used (e.g. CD4-FITC).
- [x] The axis scales are clearly visible. Include numbers along axes only for bottom left plot of group (a 'group' is an analysis of identical markers).
- [x] All plots are contour plots with outliers or pseudocolor plots.
- [x] A numerical value for number of cells or percentage (with statistics) is provided.

Methodology

Sample preparation	Cells with indicated treatment were harvested and washed once with PBS. Counting cell number and fix approx 10^6 cells with 70% ethanol overnight at 4 ºC. Centrifuge and decant ethanol thoroughly, followed by washing once with PBS. Suspend the cell pellet in 1 ml of PI staining solution (0.1% Triton X-100, 10 ug ml-1 PI, 100 ug ml-1 DNase-free RNase in PBS) and incubate in the dark at room temperature for 30 min.
Instrument	All samples were acquired on Beckman Coulter CytoFLEX LX
Software	CytExpert (v2.3)
Cell population abundance	N/A
Gating strategy	The data in the manuscript were derived from all cells without gating.

- [ ] Tick this box to confirm that a figure exemplifying the gating strategy is provided in the Supplementary Information.
